# MifM-instructed translation arrest involves nascent chain interactions with the exterior as well as the interior of the ribosome

**DOI:** 10.1038/s41598-018-28628-y

**Published:** 2018-07-09

**Authors:** Keigo Fujiwara, Koreaki Ito, Shinobu Chiba

**Affiliations:** Faculty of Life Sciences and Institute for Protein Dynamics, Kyoto Sangyo University, Motoyama, Kamigamo, Kita-Ku, Kyoto, 603-8555 Japan

## Abstract

*Bacillus subtilis* MifM is a monitoring substrate of the YidC pathways of protein integration into the membrane and controls the expression of the YidC2 (YqjG) homolog by undergoing regulated translational elongation arrest. The elongation arrest requires interactions between the MifM nascent polypeptide and the ribosomal components near the peptidyl transferase center (PTC) as well as at the constriction site of the ribosomal exit tunnel. Here, we addressed the roles played by more N-terminal regions of MifM and found that, in addition to the previously-identified arrest-provoking elements, the MifM residues 41–60 likely located at the tunnel exit and outside the ribosome contribute to the full induction of elongation arrest. Mutational effects of the cytosolically exposed part of the ribosomal protein uL23 suggested its involvement in the elongation arrest, presumably by interacting with the extra-ribosomal portion of MifM. *In vitro* translation with reconstituted translation components recapitulated the effects of the mutations at the 41–60 segment, reinforcing the importance of direct molecular interactions between the nascent chain and the ribosome. These results indicate that the nascent MifM polypeptide interacts extensively with the ribosome both from within and without to direct the elongation halt and consequent up-regulation of YidC2.

## Introduction

Living organisms have developed a variety of mechanisms to control gene expression in response to environmental and physiological changes. Regulatory nascent polypeptides provide unique feedback mechanisms to sense changes in the concentration of a metabolite or to monitor the activity of a protein localization machinery. The most salient feature of this class of regulatory factors is that they undergo regulated translation arrest^1^. *Bacillus subtilis* MifM, one of such factors, is a monitoring substrate of the YidC membrane protein biogenesis machinery and controls the expression of a YidC homolog, YidC2 (YqjG), to maintain the cellular capacity of the YidC pathways of membrane protein biogenesis^2,3^.

The YidC/Oxa1/Alb3 protein family and its distantly related EMC3, TMCO1, Get1 and Ylp1 proteins comprise a superfamily of integral membrane proteins that are conserved from bacteria to human^4–9^. They have important cellular functions of assisting membrane protein biogenesis along with the Sec machinery, which provides a major pathway of protein translocation across and insertion into the membrane^10–12^. In the bacterial Sec system, the membrane proteins SecY, SecE, and SecG, form a protein-conducting channel, called the translocon, in the cytoplasmic membrane. Translocation of secretory proteins or large extracytoplasmic domains of membrane proteins through the translocon requires SecA and SecDF^13,14^. SecA, energized by ATP, drives the polypeptide movement into the membrane^15^ and SecDF, utilizing the proton-motive force, pulls out the substrate^16–18^. YidC cooperates with the Sec machinery to assist in lipid phase integration and folding/assembly of membrane proteins^19^. Remarkably, YidC also functions in a Sec-independent manner as a membrane protein “insertase” to mediate insertion of a class of membrane proteins into the membrane^5,6^.

*B. subtilis* has two YidC homologs. SpoIIIJ (YidC1), the primary YidC factor, is constitutively expressed and YidC2, the secondary factor, is normally repressed but inducible when the SpoIIIJ activity declines^2,20^. This regulation of YidC2, fed back by the total activity of the YidC pathways^3^, features regulated translation arrest of *mifM*, the upstream open reading frame of *yidC2*. The *mifM-yidC2* mRNA can form a stem-loop secondary structure in the intergenic region, which sequesters the Shine-Dalgarno (SD) sequence required for the translation initiation of *yidC2*. Therefore, the synthesis of YidC2 is down-regulated unless the stem-loop structure is disrupted. Notably, prolonged disruption of the secondary structure and, hence, active translation of *yidC2* are brought about by the stall of the *mifM*-translating ribosome at a few codons upstream of the stop. The *mifM* open reading frame consists of 95 codons, the 5′ region of which (codons 10–34) encodes the predicted transmembrane (TM) segment, which is followed by the C-terminal cytosolic region (residues 35–95). Its translation is subject to elongation arrest at multiple sites^21^, predominantly at codons 89, 90, 91 and 92, which enhances the YidC2 expression. Importantly, MifM itself is a substrate of the YidC insertase, thus called a monitoring substrate; its engagement in the membrane insertion event results in the cancellation of the elongation arrest and consequent elongation resumption. Thus, the active membrane insertion activity of YidC in the cell leads to only a temporary elongation arrest and a repressed expression level of YidC2, whereas the reduced YidC activity of the cell leads to a prolonged MifM arrest and consequent induction of YidC2. The ability of MifM to maintain the capacity of the indispensable YidC pathway contributes to the survival of this organism under changing environmental and physiological conditions. Similar mechanisms involving regulated elongation arrest of monitoring substrates are known to operate in other bacterial species. *Escherichia coli* SecM^22,23^ and *Vibrio alginolyticus* VemP^24^ regulate the synthesis of SecA and SecD2-SecF2, respectively, according to cellular activities of protein secretion. MifM, SecM, and VemP all have arrest-evoking amino acid sequences at their C-terminal regions. However, these sequences do not share any notable similarity.

Previous genetic and structural studies of MifM showed that the elongation arrest of MifM requires interactions between the MifM nascent chain and the ribosomal components at multiple sites, from the proximity of the peptidyl transferase center (PTC) to the central constriction of the polypeptide exit tunnel. Systematic mutagenesis, such as alanine-scanning, of the C-terminal region of MifM allowed us to identify several crucial residues that reside near the PTC-proximal and mid-tunnel regions^2,21^. Also, analysis involving internal deletions of the hairpin loops of the ribosomal proteins uL4 and uL22, which contribute to the formation of the tunnel constriction, suggested that they are also important for the elongation arrest^25^. In the cryo-EM structures, the MifM nascent chain assumes an extended conformation in the exit tunnel, contacting the components at the constricted region of the tunnel and the PTC-proximal region. In particular, Glu88 contacts the PTC-forming nucleotides of the ribosomal RNA, resulting in an interference with the conformational changes of the A-site forming ribosomal RNA residues to accommodate the incoming aminoacyl-tRNA^25^.

While these studies establish the importance of the PTC-proximal and the mid-tunnel regions of the nascent MifM in the elongation arrest, the more N-terminal MifM residues were not visible in the cryo-EM structure and their roles remain to be investigated. Here, we addressed this question and found that an N-terminal region is also required for the efficient elongation arrest. Evidence suggests that extra-ribosomal interactions occur directly between MifM and the ribosomal outer surface components. Thus, we provide a unique example of arrest mechanism that involves extensive interactions between nascent chain and the ribosome both inside and outside of the ribosome.

## Results

### Frameshift analysis of the extra-ribosomal region suggests its involvement in the MifM elongation arrest

**Figure 1:**
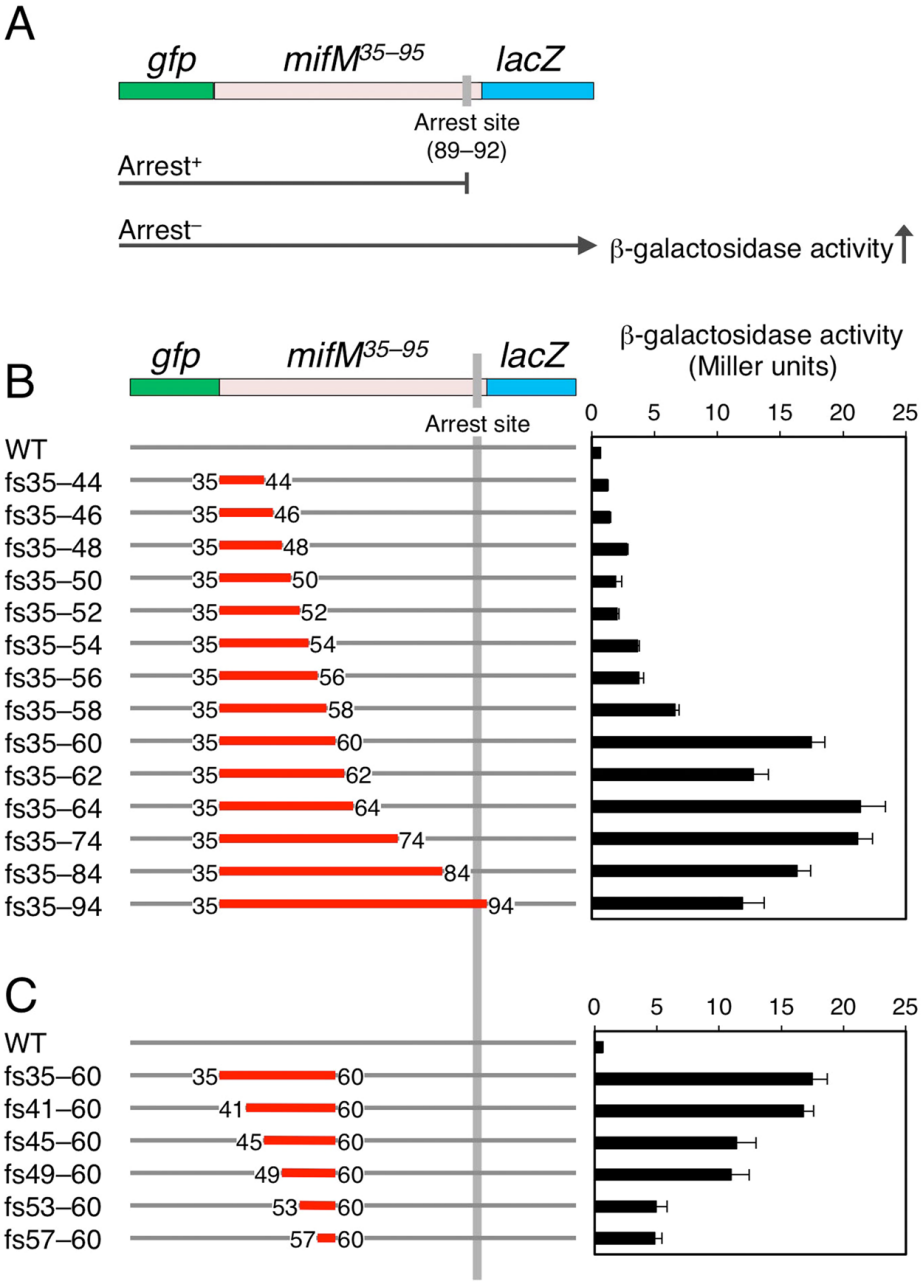
Internal frameshift mutations in the arrest site-distal region of MifM alleviate the elongation arrest. (**A**) A schematic representation of the *gfp–mifM–lacZ* fusion gene, an “arrest reporter”. The three coding regions are fused in frame such that elongation arrest at *mifM* abolishes the *cis*-encoded β-galactosidase activity. (**B**,**C**) Effects of frameshift mutations within *mifM* on the β-galactosidase activities. The frameshifted segments are indicated in red with numbers indicating the first and the last frameshifted codons. The right panels show β-galactosidase activities (mean ± s.d., n = 3) of *B. subtlis* cells expressing a reporter gene indicated in the left panels.

Translational elongation arrest of MifM can be observed clearly *in vivo* either when its N-terminal membrane-inserting segment (residues 1–34) is deleted, or when the cellular YidC insertase (SpoIIIJ) is mutationally impaired. Alternatively, it is observed in *in vitro* translation reactions, which lack the insertase. Translation of the *mifM* open reading frame of 95 codons is arrested predominantly at positions 89, 90, 91 and 92^21^. Previous genetic analysis using alanine-scanning mutagenesis targeted to residues 59–89 identified several amino acid residues, in a segment spanning from R69 to A90, as elements important for the elongation arrest^2^. The importance of the following acidic residues has also been shown^21^. However, the N-terminal boundary of the “arrest sequence” has not unequivocally been determined. To address whether a MifM region N-terminal to R69 also contributes to the arrest, we introduced a series of internal frame-shift mutations into the MifM part of the GFP-MifM-LacZ tripartite fusion protein, having MifM residues 35–95^25^ (Fig. 1A). The MifM part of the fusion protein lacks the membrane-spanning region, making it undergo constitutive elongation arrest, preventing the ribosome from reaching the LacZ-encoding region and resulting in a low level of β-galactosidase enzymatic activity. Any mutation that impairs the arrest-inducing ability of the nascent chain would allow more ribosomes to translate the *lacZ* sequence and thereby increase the β-galactosidase activity. Thus, the fusion protein can be used as a *cis* reporter of the elongation arrest efficiency at MifM. The internal −1 frameshifts are indicated by fsX-Y (e.g., fs35–95), where “X” indicates the codon, by the original *mifM* numbering, from which the first nucleotide was deleted, and “Y” indicates the codon, to which the deleted nucleotide is added back to resume the normal reading frame thereafter. Thus, the amino acid sequence of the indicated segment (in the above case, MifM residues 35–95) of GFP-MifM-LacZ is now unrelated to the wild-type MifM sequence. The *lacZ* fusion genes were integrated into the chromosomal *amyE* locus of *B. subtilis*, and β-galactosidase activities were determined for the transformants. Consistent with the previous results^25^, the fusion protein with the wild-type (WT) *mifM* sequence exhibited very low β-galactosidase activity (0.6 Miller units; Fig. 1B). By contrast, the fs35–94, fs35–84 and fs35–74 derivatives exhibited elevated β-galactosidase activities of more than 10 Miller units. These results are as expected from the previous identification of a number of arrest-important amino acid residues within the 69 to 79 interval, corresponding to the nascent chain path from the mid-tunnel region to the PTC proximity region^2^.

However, we also obtained some unexpected results with the shorter frameshifts, such as fs35–60, fs35–62 and fs35–64, having sequence alterations only in the region N-terminal to R69. Cells expressing these frameshift mutations possessed more than 10 units of β-galactosidase (Fig. 1B) even though they retained the wild-type amino acid sequence for the position 65 and its C-terminal region of MifM. Furthermore, frameshifts of even shorter N-terminal segments elevated the β-galactosidase activity (e.g. fs35–58 and others; Fig. 1B) partially. These results suggest that the arrest site-distal region of MifM contributes to the stable elongation arrest.

The almost fully arrest-defective phenotype of fs35–60 indicated that the segment 35–60 contained some arrest-essential element. To further delineate such a determinant, we constructed another series of frameshifts ending at the 60th codon of *mifM* (Fig. 1C). The fs41–60 frameshift elevated β-galactosidase activities almost as potently as fs35–60 (Fig. 1C). Shorter frameshifts, fs45–60 fs49–60, fs53–60 and fs57–60, gave weaker but significant effects; the former two gave intermediate levels, and the latter two gave still lower levels of β-galactosidase (Fig. 1C). Thus, the highest level of β-galactosidase and, hence, the complete abolishment of the elongation arrest required the alteration of the 41–60 segment, although the gradual effects of the shorter frameshifts indicated the absence of a clear cut-off point. The results so far obtained suggest that the upstream ~20 amino acids of residue 60 contribute to the efficient elongation arrest of MifM.

**Figure 2:**
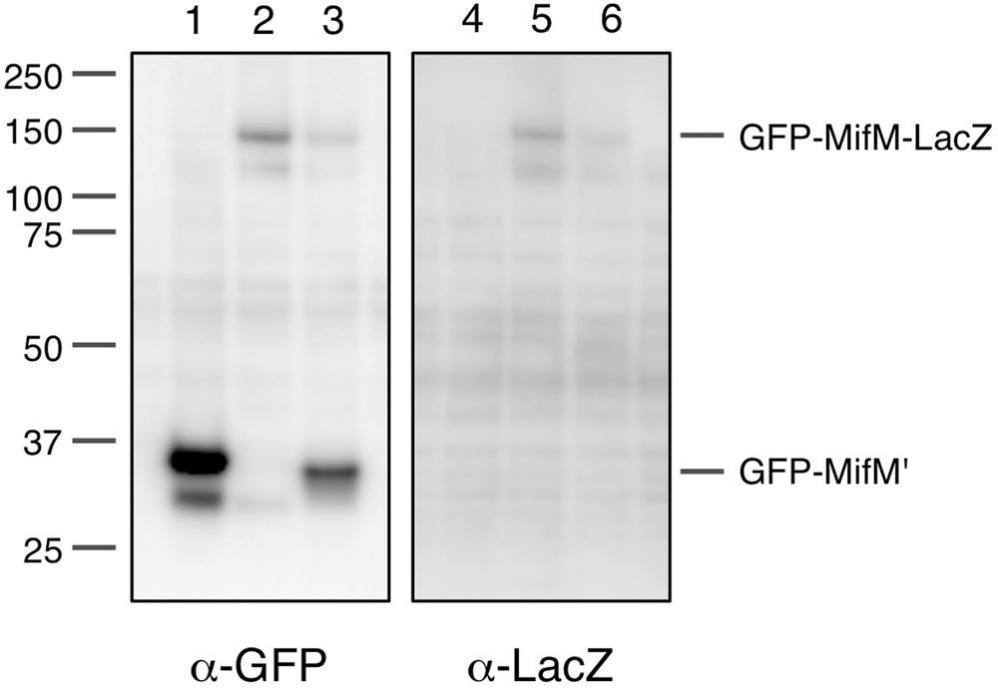
Electrophoretic visualization of the *in vivo* translation products. The *gfp-mifM-lacZ* gene fusion (lanes 1 and 4) and its mutant forms, fs41–60 (lanes 2 and 5) and GS41–60 (lanes 3 and 6), were expressed in *B. subtilis*, and the arrested (GFP-MifM’) and the full-length (GFP-MifM-LacZ) translation products were detected by anti-GFP (lanes 1–3) and anti-LacZ (lanes 4–6) immunoblotting, respectively, after separation by SDS-PAGE. The tRNA moiety of the arrest product had been removed under the sample preparation conditions used. Full-length blots are presented in Supplementary Fig. 2A.

We examined the effect of the fs41–60 frameshift on the production of the GFP-MifM-LacZ fusion protein by Western blotting experiments (Fig. 2). As expected from our previous study^2^, the wild-type GFP-MifM-LacZ protein was produced in the cell predominantly as the arrested product (GFP-MifM’) that migrated slightly faster than the 37 kDa molecular mass marker (note that tRNA had been removed from nascent peptidyl-tRNA under the experimental conditions used). We did not detect any band of the full-length product of the predicted molecular mass of 153 kDa by anti-GFP or anti-LacZ immunoblotting (Fig. 2, lanes 1 and 4). By contrast, we detected a ~150 kDa band for the fs41–60 derivative of the fusion protein with anti-GFP and anti-LacZ (Fig. 2, lanes 2 and 5), but not any arrest product (Fig. 2, lane 2). Thus, the frame-shifted amino acid sequence at the 41–60 segment of MifM allows the ribosome to continue translation beyond the MifM region. In other words, the elongation-arresting ability of MifM was abolished by the frameshift mutation. The region upstream of residue 60 plays an important role in the MifM elongation arrest.

### Disorder introduction analysis of the extra-ribosomal region suggests its involvement in the MifM elongation arrest

The exit tunnel of the ribosome can accommodate 30–40 amino acid residues of a nascent polypeptide^26^. Given that the elongation-arrested MifM nascent chain assumes an extended conformation in the tunnel as shown by the cryo-EM structures^25^, the MifM residue 60, which is ~30 residues apart from the arrested ends (residues 89–92), is likely to reside in the vestibule region to the exit port of the ribosome. It follows then that the more N-terminal region has already exited from the tunnel and is exposed to the cytosol.

The above results that elongation arrest is compromised by the fs41–60 mutation could be interpreted in at least two different frameworks; loss of function and gain of function. The loss-of-function possibility assumes that the wild-type 41–60 segment has an essential role in inducing the full elongation arrest, which is abolished by the fs41–60 alteration. The gain-of-function possibility assumes that the frame-shifted amino acid sequence somehow acts to cancel the elongation arrest of the MifM nascent polypeptide, which is brought about by the actions of the C-terminal determinants. For instance, the altered sequence could induce a local folding of the nascent chain around the tunnel exit region, which in turn generates a mechanical pulling force against the nascent chain-ribosome complex to trigger release from the arrest. This possibility is conceivable because the elongation arrest of *E. coli* SecM can be canceled by inserting a folding domain into a nascent chain region around the tunnel exit^27–31^.

**Figure 3:**
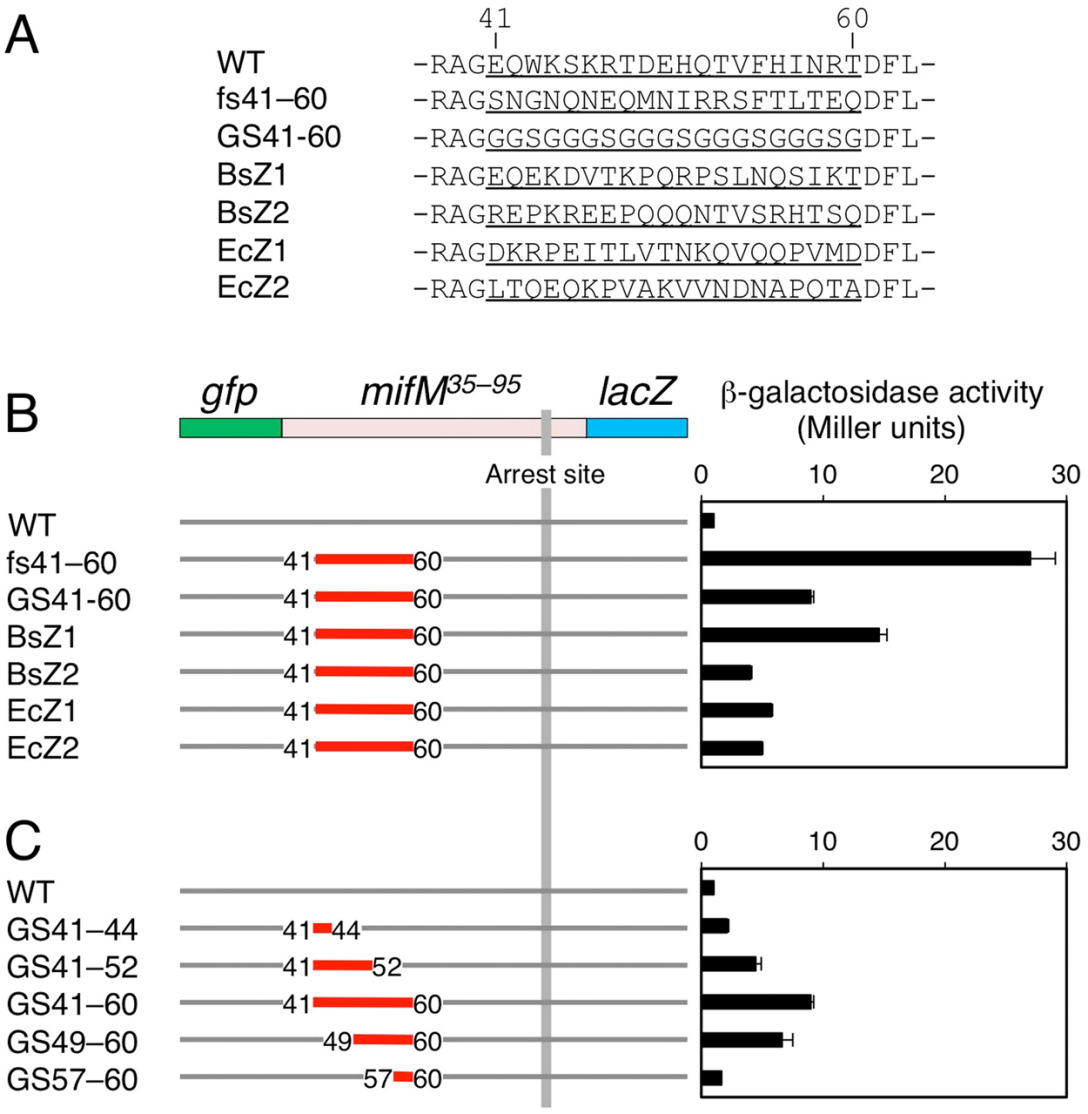
Unstructured sequences at the 41–60 segment of MifM alleviate the elongation arrest. (**A**) Amino acid sequences of the MifM residues 41–60 (with 3 preceding and following residues as well) and those of the corresponding positions in the mutant versions of *gfp–mifM–lacZ*. The GS41–60 mutant has five repeats of GGSG. BsZ1 and BsZ2 have the amino acid sequences 317–226 and 344–363 of *B. subtilis* FtsZ, respectively, whereas EcZ1 and EcZ2 have the sequences 318–337 and 347–366 of *E. coli* FtsZ, respectively. (**B**,**C**) Effects of the internal substitutions of *mifM* on the β-galactosidase activities. The sequence substitutions are indicated in red with numbers indicating the first and the last amino acids of the substitutions. The right panels show β-galactosidase activities (mean ± s.d., n = 3) of *B. subtlis* cells expressing a fusion gene indicated in the left panels.

As a means to distinguish the two possibilities, we replaced the MifM region N-terminally adjacent to residue 60 by amino acid sequences of low folding propensities. We used 5 × [GGSG]^32^, as well as sequences derived from intrinsically disordered regions, BsZ1, BsZ2, EcZ1 and EcZ2, of the *B. subtilis* and the *E. coli* FtsZ proteins^33^ (Fig. 3A) to substitute for the MifM segment 41–60 in the GFP-MifM-LacZ fusion protein (Fig. 3B). All of the variants thus constructed were found to give higher β-galactosidase activities than the control protein with the wild-type MifM sequence. The extents of activity elevation differed for the different mutants. The fs41–60 derivative exhibited the highest β-galactosidase activity (27.0 units), and the BsZ2 exhibited the lowest activity (4.0 units).

The 5 × [GGSG] mutant (GS41–60) gave β-galactosidase activity (9.0 units) that was about 1/3 of the activity observed with the fs41–60 mutant. *In vivo* accumulation of GS41–60 was examined by anti-GFP and anti-LacZ Western blotting (Fig. 2). Anti-GFP detected both the full-length (GFP-MifM-LacZ) and the arrested (GFP-MifM’) products of the GS41–60 form of GFP-MifM-LacZ (Fig. 2, lane 3), whereas anti-LacZ detected the full-length GS41–60 product (lane 6). Comparing to the wild-type fusion protein (lanes 1 and 4), the proportion of the arrested product decreased and that of the full-length product increased. These results establish that introduction of five GGSG repeats into position 41–60 of MifM compromises the elongation arrest, albeit less pronouncedly than the original frameshift mutation. Comparison of mutants having different numbers (1, 3 or 5) of [GGSG] units in the 41–60 segment showed that the β-galactosidase up-regulating effects were stronger for mutations introducing longer repeats (Fig. 3C). Thus, replacements of the 41–60 segment with different classes of unrelated and putatively unstructured sequences invariably impaired, although partially in most cases, the elongation-arresting function of MifM, supporting the notion that this region of MifM positively contributes to the stable elongation arrest.

### *In vitro* translation analysis further supports the involvement of the extra-ribosomal region in the MifM elongation arrest

**Figure 4:**
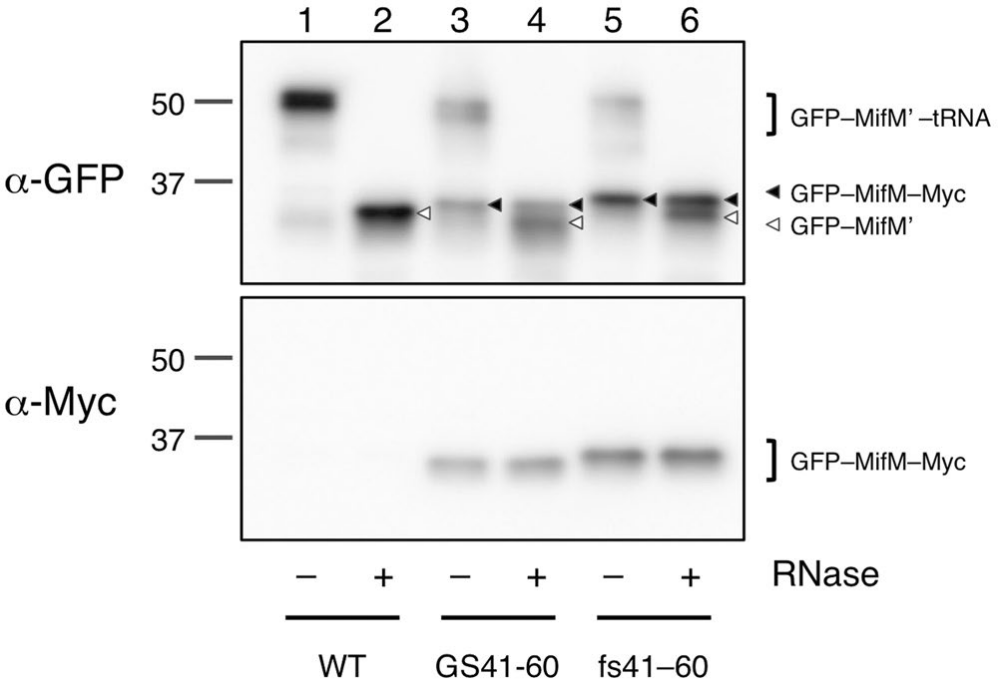
Mutations of MifM residues 41–60 compromise elongation arrest *in vitro*. *In vitro* translation using *Bs* hybrid PURE system was directed by the *gfp–mifM*^35–95^*–myc* templates with or without the *mifM* mutations affecting residues 41–60, indicated at the bottom. Reaction products were analyzed by SDS-PAGE using the neutral pH (WIDE-RANGE) gel system, followed by immunoblotting with anti-GFP (upper panel) and anti-Myc (lower panel). To remove the tRNA moiety from polypeptidyl-tRNA, portions of samples were treated with RNaseA before electrophoresis (lanes with even-numbers). Black and white triangles indicate full-length (GFP-MifM-Myc) and arrested (GFP-MifM’) forms of the translation product, respectively. Full-length blots are presented in Supplementary Fig. 2B.

It is important to address whether the mutational effects observed *in vivo* have resulted from the defective function of some specific factor that interacts with the arrest site-distal region of MifM. We showed previously that elongation arrest of MifM was recapitulated *in vitro* using *Bs* hybrid PURE system^34^ composed of the purified translation components from *E. coli* and the ribosomes from *B. subtilis*. Although this previous result makes the involvement of a cytosolic factor unlikely, *in vitro* analysis in the present framework of study should be useful. Thus, we studied translation of GFP-MifM-Myc as well as its GS41–60 and fs41–60 variants in the *Bs* hybrid PURE system. *In vitro* translation was programmed with the wild-type and the mutant DNA templates and translation products were separated by SDS-PAGE at neutral pH for detection of the products with anti-GFP and anti-Myc immunoblotting. Translation of wild-type *gfp-mifM-myc* for 30 minutes at 37 °C produced a major product that migrated at a position of ~50 kDa, which was detectable with anti-GFP but not with anti-Myc (Fig. 4, upper panel, lane 1). After RNase treatment of the sample, the 50 kDa band disappeared, which instead migrated at the position slightly below the 37 kDa molecular mass marker (Fig. 4, upper panel, lane 2). Thus, the 50 kDa and the ~37 kDa bands should have represented the elongation-arrested polypeptidyl-tRNA (GFP-MifM’-tRNA) and its polypeptide moiety (GFP-MifM’), respectively. The full-length protein (see below) was not appreciably detected (Fig. 4, lanes 1 and 2, upper and lower panels), confirming that *in vitro* translation of *mifM* in *Bs* PURE system is subject to efficient elongation arrest^34^.

By contrast, *in vitro* translation of the GS41–60 derivative produced the full-length product (GFP-MifM (GS41–60)-Myc) that was reactive both with anti-GFP and anti-Myc, along with a reduced amount of the arrested polypeptidyl-tRNA (Fig. 4, lanes 3 and 4). The fs41–60 derivative produced similar products as GS41–60, with a still increased proportion of the full-length species and a decreased level of arrest product (Fig. 4, lanes 5 and 6). The stronger defect with the fs mutation than the GS mutation is in accordance with the *in vivo* results. These results establish that altered 41–60 sequences of MifM impair the ability of MifM to undergo stable elongation arrest *in vitro*. Importantly, the mutational effects did not involve any non-ribosomal *B. subtilis* proteins. Although the essential translation factors from *E. coli* were present in the reaction, it was unlikely that they participated in the species-specific arrest of MifM^34^. Taken together, these results are consistent with the notion that the extra-ribosomal region of MifM directly interacts with the translating ribosome to materialize the effective elongation arrest.

### Truncation analysis of the extra-ribosomal region demonstrates its indispensability in the MifM elongation arrest

**Figure 5:**
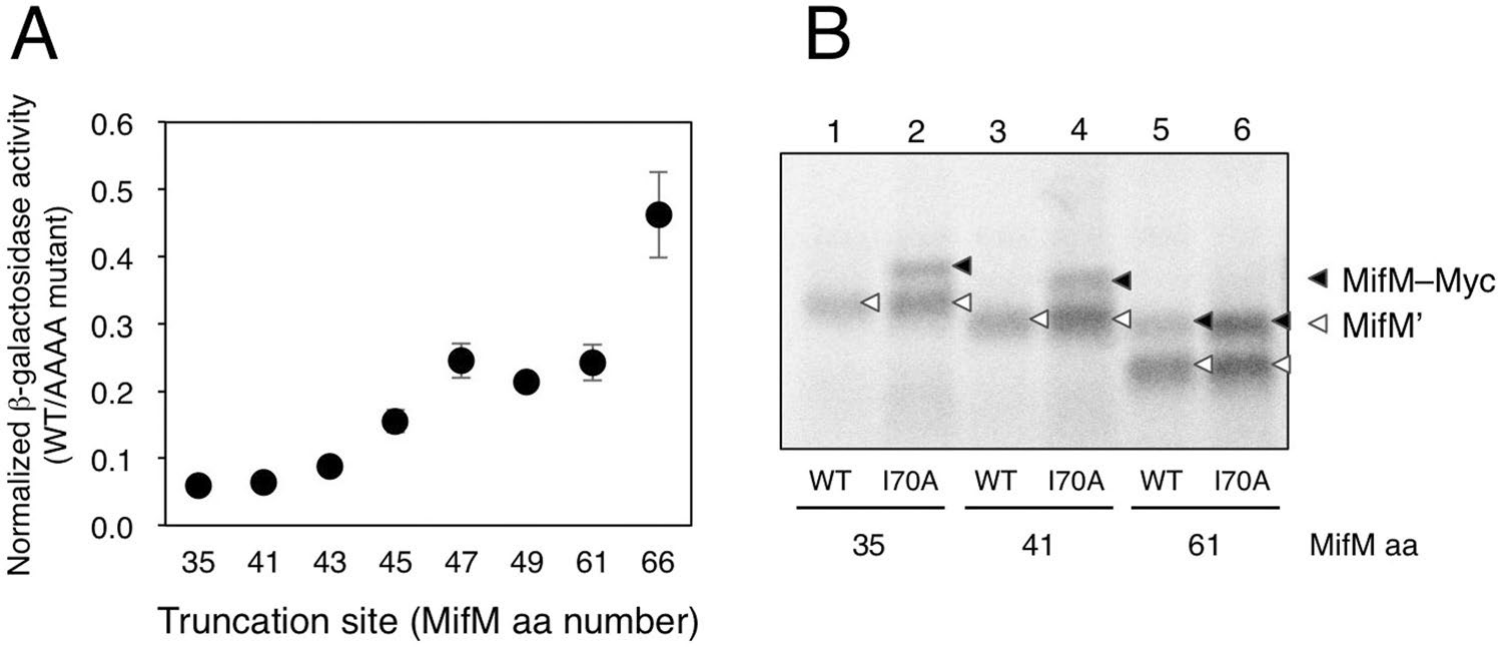
The absence of the MifM region N-terminal to residue 60 compromises the elongation arrest *in vivo* and *in vitro*. (**A**) Arrest efficiencies of MifM mutants lacking N-terminal sequences. β-galactosidase activities (mean ± s.d., n = 3) of cells harboring *Pgrac-mifM-lacZ* derivatives with the N-terminal truncations of MifM, with or without additional arrest-impairing mutation (AAAA) at the PTC-proximal region, were measured. The ratio of β-galactosidase activity (WT/AAAA) is shown for each N-terminal mutation. The numbers below the graph indicate the first MifM residues in the truncated MifM mutant derivatives (numbering by the wild-type MifM protein) (**B**) *In vitro* translation of MifM mutants lacking N-terminal sequences. Similar to (**A**), each N-terminal mutation was combined with a mutation I70A at a mid-tunnel region, which impairs the arrest partially (this mutation was used as it did not affect the electrophoretic mobility of MifM). DNA templates, with the N-terminal truncations indicated by the residues that now became the first *mifM*-encoded residues, were used to direct *in vitro* translation with *Bs* hybrid PURE system in the presence of ^35^S-methionine. Samples were treated with RNase A, separated by SDS-PAGE and radioactive products were analyzed with a phosphorimager. Black and white triangles indicate full-length (MifM-Myc) and arrested (MifM’) forms of the translation products, respectively. Full-length gel is presented in Supplementary Fig. 2C.

Although the *in vivo* and *in vitro* experiments suggest that the MifM residues 41–60 is required for the efficient elongation arrest, in theory, they have a limitation of relying on sequence alterations. A straightforward way to address the essentiality of that region would be to examine effects of the simple absence of the sequence. Thus, we addressed whether deletion of this region compromised the elongation arrest. For *in vivo* analysis, we fused N-terminally truncated variants of MifM in-frame to the N-terminal region of LacZ. We retained the first two *mifM* codons in all the constructs to minimize variation in the translation initiation efficiencies. To assess elongation arrest-dependent decline in the β-galactosidase expression from the fusion protein, we also constructed their arrest-defective versions by introducing the quadruple alanine substitution mutation at the PTC-proximal residues 86–89 (AAAA mutant), which was shown previously to abolish the arrest^21^. We measured β-galactosidase activities in sets of cells expressing “wild-type” and the AAAA mutant versions of the fusion protein. Because the sequence alterations at the regions that immediately follow the start codon caused variations of translation yields due presumably to altered initiation efficiencies among different MifM derivatives, we used WT/AAAA ratio of β-galactosidase activity as a quantitative indication of arrest efficiency, referred to as “normalized β-galactosidase activity”. Such normalized values allowed us to compare different constructs with respect to the extents of elongation arrest even though the manipulations of the N-terminus coding regions had affected the translational initiation efficiencies. The MifM-LacZ reporter containing the MifM residues 35–95 (MifM^35–95^-LacZ) showed the normalized β-galactosidase activity of 0.06 (Fig. 5A). Deletion of the MifM residues 35–40 (MifM^41–95^-LacZ) still gave a low normalized β-galactosidase activity (0.06), comparable with that of MifM^35–95^-lacZ, indicating that residues 35–40 play little, if any, role in the elongation arrest of MifM. Further deletions up to residue 46 gradually increased the normalized β-galactosidase activities, from 0.09 in MifM^43–95^-LacZ to 0.25 in MifM^47–95^-lacZ (Fig. 5A). Truncations beyond residue 47 gave the high normalized β-galactosidase activity of 0.2 or higher, for example, 0.24 for MifM^61–95^-LacZ, which lacks the N-terminal region of MifM up to residue 60. These results indicate that the absence of the N-terminal segment compromised the elongation arrest and therefore the N-terminal segment has a positive role in the arrest, substantiating our conclusion obtained from the sequence alteration experiments. From the Fig. 5 results, we can surmise that the full arrest determinant (arrest sequence) starts at residue 41.

We also investigated translation of the N-terminally truncated constructs of MifM-Myc *in vitro*, using the *Bs* hybrid PURE system in the presence of ^35^S-methionine. To enhance the radiolabeling, we introduced two additional methionine codons just after the initiation codon used for translation of the truncated *mifM*-coding sequences. Labeled translation products were treated with RNase A to remove tRNA moieties of peptidyl-tRNA and then analyzed by SDS-PAGE followed by autoradiography. *In vitro* translation of *mifM*^*35–95*^*-myc* produced a single band, which likely represented the arrested product. This assignment was supported by comparison with the product pattern obtained with the same construct but having the Ile70Ala mutation, which partially alleviated the arrest^2^. The latter construct produced two bands, the arrested fragment and the full-length fusion protein (Fig. 5B, lanes 1 and 2). Essentially the same results were obtained for *mifM*^*41–95*^*-myc* and its I70A derivatives (lanes 3 and 4). These results indicate that the *mifM*^35–95^*-myc* and *mifM*^41–95^*-myc* underwent efficient elongation arrest such that the full-length products were not appreciably produced, unless the partially arrest-alleviating I70A mutation was introduced. By contrast, translation of *mifM*^61–95^*-myc* produced both the arrested and the full-length polypeptides even without introducing the I70A mutation (Fig. 5B, lanes 5 and 6), indicating that the arrest efficiency was reduced when MifM lacked the residues 41–60. Taken together, these *in vivo* and *in vitro* results establish that the efficient elongation arrest of MifM requires residues 41–60 in addition to the previously identified arrest-important amino acid sequence located in the mid-tunnel to the PTC region of the ribosome.

### Roles of ribosomal surface components in the elongation arrest of MifM

**Figure 6:**
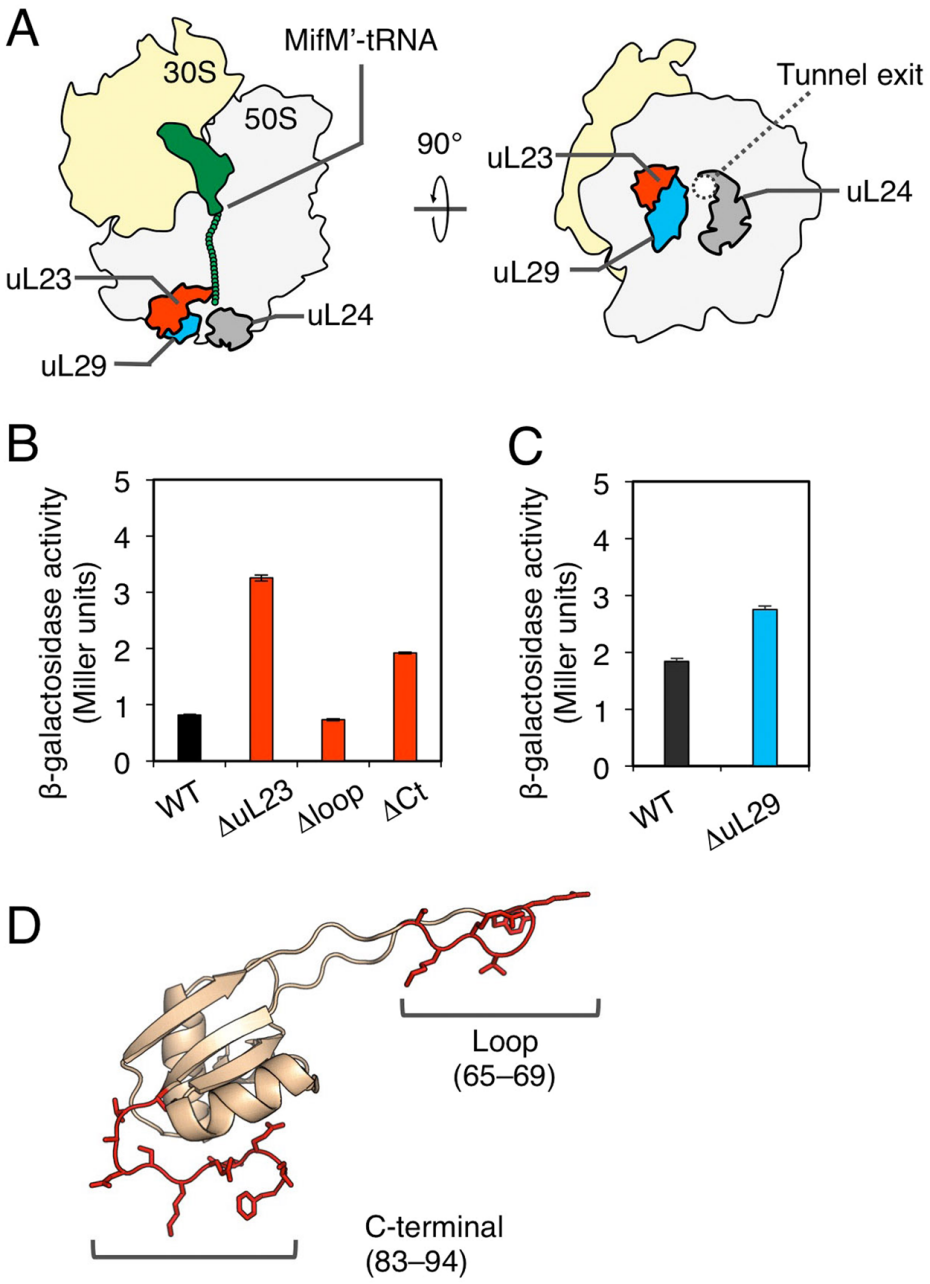
Effects of uL23 and uL29 mutations on the MifM elongation arrest (**A**) Schematic illustrations of the bacterial ribosomal proteins near the tunnel exit. (**B**,**C**) β-galactosidase activity (mean ± s.d., n = 3) of the GFP-MifM-LacZ fusion protein, expressed in wild type (WT) or the indicated ribosomal protein mutants. ΔuL23 and ΔuL29 indicate total deletion of the *rplW* encoding uL23 and *rpmC* encoding uL29, respectively. Δloop and ΔCt indicate internal deletions of uL23, lacking residues 65–69 and 83–94, respectively. (**D**) Structure of uL23 (PDB ID: 3J9W). The loop and C-terminal regions are colored in red.

Our results raise an intriguing possibility that the MifM nascent chain interacts with the surface of the ribosome to fully exert its elongation arrest function. In this case, mutations affecting the putative interaction partner on the ribosome could also compromise the elongation arrest. The tunnel exit of the ribosome is surrounded by the surface-exposed regions of ribosomal proteins uL23, uL24 and uL29 (Fig. 6A). We constructed *B. subtilis* mutants deleted for *rplW* (ΔuL23) or *rpmC* (ΔuL29), both of which are non-essential for the growth of this bacterium^35^. Reporter assays using *gfp-mifM-lacZ* revealed that the ΔuL23 mutant exhibited three times higher activity of β-galactosidase than the wild-type control (Fig. 6B). The intra-ribosomal region of uL23 forms a loop that protrudes into the exit tunnel (Fig. 6A). As reported previously^25^, the internal deletion of the uL23 residues 65–69, which forms the intra-tunnel loop (Fig. 6D), did not appreciably compromise the elongation arrest, thus giving low β-galactosidase activity (Fig. 6B, Δloop). By contrast, the internal deletion of residues 83–94 at the C-terminal tail of uL23, which is exposed to the ribosomal surface (Fig. 6D), elevated the β-galactosidase activity (Fig. 6B, ΔCt) partially. The ΔuL29 mutation also elevated the β-galactosidase activity albeit to a lesser extent than the ΔuL23 mutation (Fig. 6C). These results revealed that alterations of some ribosomal surface components lead to decreased ability of the MifM nascent polypeptide to exert stable elongation arrest. We suggest that the effective elongation arrest of MifM requires interactions between the MifM nascent chain and the ribosomal surface components near the exit port.

## Discussion

A key feature of regulatory arrest peptides is that they have options of arresting translation stably or releasing the arrest under specific conditions to respond to environmental or physiological changes. Whereas a class of them, having chemical ligand-dependent arrest mechanisms, respond to antibiotics or metabolites, another class of regulatory nascent chains has an intrinsic arrest mechanism, which is combined with an arrest-releasing mechanism. The latter class of arrest peptides is also a substrate of a protein localization machinery such that the engagement of its nascent chain in the protein localization process, through the N-terminal topogenic sequence, leads to release from the arrest^36^. Thus, detailed knowledge about the ribosome-stalling molecular interactions would be a prerequisite for our understanding of the remarkable mode of regulation featuring a monitoring substrate as an environmental sensor.

We have shown here that MifM entails not only the previously identified arrest-essential amino acid residues encoded by the C-terminal quarter of the coding sequence but also residues 41–60 located more N-terminally as the determinant of elongation arrest. It is likely that the latter segment is, at least in part, exposed to the surface of the ribosome, raising a possibility that the MifM nascent chain interacts with the tunnel vestibule and/or outer surface components in addition to those at the PTC and the exit tunnel. Indeed, the uL23 mutant deleted for the surface-exposed region partially compromised the elongation arrest (Fig. 6). By contrast, the uL23 mutant deleted for the intra-tunnel loop did not affect the arrest efficiency (Fig. 6). Therefore, it was unlikely that the deletion of the surface-exposed region allosterically affected the uL23′s intra-tunnel region that may have actually been required for the arrest. These data support the notion that MifM interacts with the ribosome surface facing the cytosol. We further reproduced the elongation arrest of MifM in the *in vitro* translation reaction using *Bs* PURE system, excluding the involvement of any non-ribosomal factor in the arrest induction and thus supporting the above conclusion.

**Figure 7:**
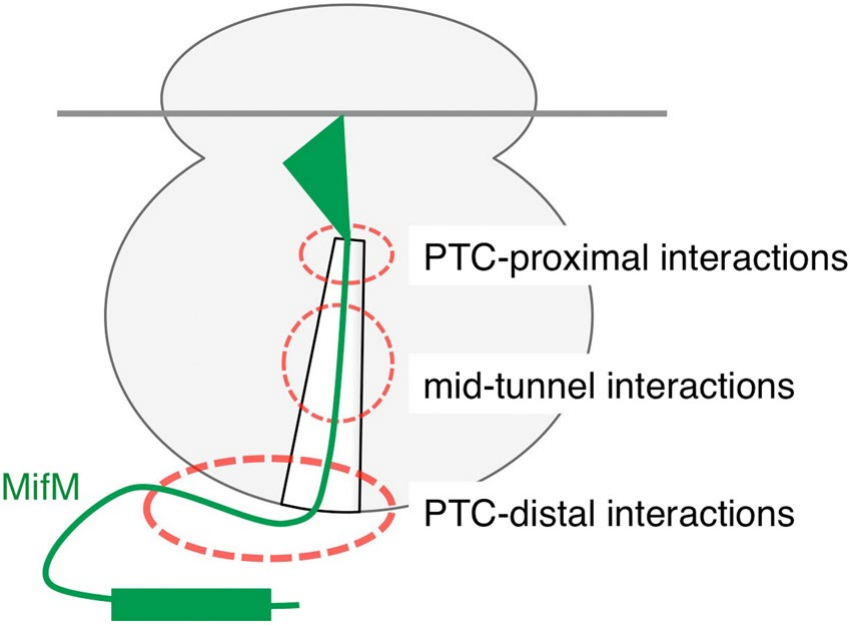
A schematic illustration showing that the MifM nascent chain interacts extensively with the ribosome to fully induce the elongation arrest.

Our previous and current results indicate that translation arrest of MifM is accomplished by at least three categories of interactions between the nascent polypeptide and the ribosome: interactions involving components within or in the close proximity of the PTC, interactions at the mid-tunnel region and interactions beyond the tunnel exit, likely involving ribosomal exterior components such as the surface-exposed domains of the uL23 and uL29 proteins (Fig. 7). Thus, the MifM nascent chain interacts extensively with the ribosome to stall it before termination. Apart from the N-terminal membrane insertion sequence, the amino acid sequence of MifM seems to be dedicated to the prevention of its translation from completion.

While we uncovered the roles played by the 41–60 region of MifM in the elongation arrest, the proposed extra-ribosomal interactions may not be as specific as the interactions involving the intra-ribosomal MifM sequence. Alanine-scanning mutagenesis experiments did not yield any single amino acid substitution mutation in this region that impaired the MifM’s ribosome-stalling activity (Supplementary Fig. S1), whereas several residues at the 69–79 interval had been identified as specifically required for the arrest^2^. Sequence alterations of different short segments of the 41–60 region gave moderate arrest defects (Figs 1C, 3C and 5A). Also, the arrest defects due to the absence of uL23 or uL29 were partial (Fig. 6). From these observations, we suggest that the extra-ribosomal region of the MifM nascent chain contributes to the elongation arrest through multiple but weak interactions with the ribosome. Whereas the geometrical restriction within the tunnel interior would facilitate specific interactions, extra-ribosomal segment of the nascent chain would gain more freedom to fluctuate and, hence, more promiscuous interaction properties. MifM may have developed controllable elongation arrest by combining these two modes of molecular interactions (see below).

According to the cryo-EM structural studies of MifM nascent chain-ribosome complexes, the PTC-proximal MifM residues interact with the ribosomal RNA bases to configure the PTC in a non-productive form^25^. An important question now is how the PTC-distal interactions contribute to the development of the anomalous conformation of the PTC. One obvious possibility would be that the extra-ribosomal interaction might allosterically affect the conformation of the PTC through a cascade of ribosomal conformational changes from the PTC-distal to the PTC-proximal locations of the ribosome. Although we cannot exclude this possibility, the MifM-translating ribosome did not exhibit any gloss structural difference from the normal ribosome, except for the PTC region^25^. Also, the lack of a strict sequence requirement for the extra-ribosomal section seems to disfavor the allosteric transmission theory. Less specific mechanisms of the extra-ribosomal participation in the arrest are conceivable. For instance, the extra-ribosomal interaction may simply reduce the Brownian diffusion movement of the nascent chain region exposed to the cytosol, which might otherwise destabilize the MifM-ribosome interactions in the mid-tunnel region physically. We assume that the mid-tunnel interaction would then induce somehow the PTC-interfering interaction with the critical PTC residues^25^. As a simple version of such a possibility, non-specific “sticky” interactions between MifM and the ribosomal surface might be sufficient to reduce the destabilizing mobility of the nascent chain. In this respect, it is interesting to note that replacements of the 41–60 segment of MifM with various unrelated sequences gave different impacts on the elongation arrest stability (Fig. 3). It is important to study whether electrostatic properties of the ribosomal surface^37–39^ contribute to the arrest-related extra-ribosomal interactions.

The present results raise a question about whether the extra-ribosomal sequences play roles generally in translation arrest observed in regulatory nascent polypeptides. Yang *et al*.^40^ concluded that the extra-ribosomal region of SecM has an arrest-reinforcing role, on the basis of the *in vitro* observations that sequence substitutions at the regions N-terminal to the originally determined “arrest sequence“^23^ of SecM impaired the arrest. However, earlier studies of SecM showed that SecM segments 150–166, 140–166^23^ and 120–166^41^ could effectively halt translation continuation *in vivo* and *in vitro*. Therefore, the question seems to be still open whether the extra-ribosomal region of SecM has a positive role in the elongation arrest. In this context, it is of note that the 17-residue SecM “arrest sequence” has been used extensively to detect and analyze co-translational targeting or folding of inserts placed upstream of it; the elongation arrest is indeed subject to release when the folding or membrane insertion takes place near the ribosomal tunnel exit region^27–31,42^. Thus, as already discussed in the earlier section, sequence substitutions at the N-terminal region could artificially interfere with the elongation arrest. In the present study, by contrast, we have examined the effects of the absence of the N-terminal segments, allowing us to conclude unequivocally that the extra-ribosomal region of MifM has a positive role in the elongation arrest. Finally, mutational analysis of the amino acid sequence of VemP, another “force-sensitive” regulatory nascent polypeptide^24^, suggests that the N-terminal region of this protein has a minor but definite role in the elongation arrest^43,44^.

The mechanism by which the engagement of the N-terminal transmembrane region of MifM in the YidC-dependent membrane insertion process leads to the release of elongation arrest remains unclear. The force-sensitive nature, which has well been documented by the model experiments^27^ for the arrest release of SecM, may underlie the arrest release in MifM as well. Our previous results that the replacement of the N-terminal transmembrane segment of MifM with a secretion signal sequence converted MifM to a sensor of the Sec pathway activity seems to be consistent with the physical pulling mechanism of arrest release in MifM^34^. However, given the lack of information about the intensity of the force generated by the YidC-mediated membrane insertion, modes of nascent chain-ribosome interactions could provide some clue about the important question of how the membrane insertion event triggers the arrest release in the physiological settings. If the sum of multiple and weak interactions at the ribosomal outer surface is required for the full extent of arrest, disruption of any one of them by a weak trigger would be sufficient to impair the arrest, at least partially at first but could be propagated to a full cancellation eventually by multiple triggers. Thus, the widespread MifM nascent chain-ribosome interactions can make the arrest extremely susceptible to a pulling force applied to the nascent chain. The surface events would then be transmitted to the disruption of the nascent chain-ribosome interactions at the mid tunnel region, followed by further transmission of the conformational changes to the PTC region of the polypeptide. In addition to the generation of a pulling force, the YidC machinery itself, which has a unique helix-turn helix structure protruding into the cytoplasm, may sterically disrupt the nascent chain-ribosome surface interaction upon engagement with the MifM nascent chain, thereby contributing to the arrest-releasing event. Based on these considerations, we propose that the extra-ribosomal interactions allow the elongation arrest of MifM to be receptive to regulation by the nascent chain’s engagement in the YidC-mediated membrane integration processes, which is not directly powered by energy sources, such as ATP. Clearly, further studies are required to clarify the role of the extra-ribosomal interaction in the MifM function. As local variations of elongation speed could influence folding and targeting of a newly synthesized protein^45–50^, it will be interesting to ask whether nascent chain interactions with the outer surface of the ribosome contribute to the translational pausing events that many proteins experience, as revealed by our nascent chain profiling experiments^51^.

## Materials and Methods

### Bacterial Strains and Plasmids

*B. subtilis* strains, plasmids and DNA oligonucleotides used in this study are listed in Supplementary Tables S1, S2 and S3, respectively. The *B. subtilis* strains were derivatives of PY79 (wild-type^52^) and were constructed by transformation with plasmids listed in Supplementary Table S2. Plasmids were constructed by standard cloning methods, PCR, site-directed mutagenesis^53^, PrimeSTAR mutagenesis (Takara), Gibson assembly^54^ and combinations of these methods^55^. Following introduction into the *B. subtilis* chromosome by homologous recombination, the recombinants were checked for their antibiotic resistance, inactivation of *amyE* and the absence of any drug resistance markers originally present on the plasmid backbone.

### β-galactosidase Assay

*B. subtilis* cells were cultured at 37 °C in LB medium. Samples were withdrawn from 3 mL cultures at an optical density at 600 nm (OD_600_) of 0.5–1.0 and used for β-galactosidase activity assays^20^. The kinetic β-galactosidase assay presented in Fig. 5A and Supplementary Fig. S1 was performed as described previously^56,57^ with the following modifications. *B. subtilis* cells were grown at 37 °C for 120–150 min in LB medium with (Fig. 5A) or without (Figure S1) 1 mM isopropyl-β-D-thiogalactopyranoside, after 50-fold dilution from overnight cultures and using a 96-well deep well plate equipped with a shaking device. 100 μL portions of the cultures were then transferred to individual wells of another 96-well plate for a Thermo Scientific Multiskan Go microplate spectrophotometer and OD_600_ was recorded. Cells in each well were then lysed by adding 50 μL of Y-PER reagent (Thermo Scientific), mixing thoroughly and incubating for 20 min at room temperature. For β-galactosidase assay, 30 μL of o-nitrophenyl-β-D-galactopyranoside (ONPG) in Z-buffer (60 mM Na_2_HPO_4_, 40 mM NaH_2_PO_4_, 10 mM KCl, 1 mM MgSO_4_, 38 mM β-mercaptoethanol) was added to each well. The reaction solution was mixed thoroughly and then OD_420_ and OD_550_ were measured every 5 min over 60 min at 28 °C. Arbitrary units [AU] of β-galactosidase activity were calculated by the formula [(1000 × V_420_ − 1.3 × V_550_)/OD_600_], where V_420_ and V_550_ are the first order rate constants, OD_420_/min and OD_550_/min, respectively. All comparisons were made among strains of isogenic backgrounds.

### *In vitro* Translation

The *E. coli*-based coupled transcription-translation system composed of the purified components (PUREfrex 1.0; GeneFrontier) was used for *in vitro* translation as described previously^21,34,58^ with some modifications. A preparation of the *B. subtilis* ribosomes^34^ was used at a final concentration of 1 μM. 2.5 U/μL of T7 RNA polymerase (Takara) was added further to the reaction mixture to reassure transcription. The reaction was primed with appropriate DNA templates prepared by PCR using appropriate primers (Supplementary Table S4) and allowed to continue at 37 °C for 30 min (Fig. 4). Samples were withdrawn, mixed with the same volume of 2xSDS-PAGE loading buffer (250 mM Tris-HCl (pH 6.8), 4% (wt/vol) SDS, 30% (vol/vol) glycerol, 10 mM DTT, a trace amount of bromophenol blue) and incubated at 37 °C for 10 min. When indicated, samples were further treated with 0.2 mg/ml RNase A (Promega) at 37 °C for 10 min immediately before electrophoresis. For the nascent chain labeling with ^35^S-methionine (Fig. 5B), ^35^S-methionine was added to the *in vitro* translation reaction mixture at the final isotope concentration of 9.4 MBq/mL; reaction products were treated with RNase A in SDS-PAGE loading buffer and separated by 12% Nu-PAGE Bis-Tris gels with MES-SDS running buffer (Thermo Scientific), followed by visualization of radioactive polypeptides with FLA7000 (GE Healthcare) phosphorimager.

### Western Blotting

For Western blotting, samples in SDS-PAGE loading buffer were separated by 8% or 12% gels prepared with WIDE RANGE Gel buffer (Nacalai Tasque) according to the manufacturer’s instructions, transferred onto a PVDF membrane and then subjected to immuno-detection using antibodies against GFP (A-6455; Invitrogen), LacZ (A-11132; Invitrogen), MifM^34^, or c-Myc (A-14; Santa Cruz Biotechnology) as described previously^34^. Images were obtained and analyzed using Amersham Imager 600 (GE Healthcare) luminoimager.

## Electronic supplementary material

Supplementary Information
