# Supplementary material for: MifM-instructed translation arrest involves nascent chain interactions with the exterior as well as the interior of the ribosome
Source: Sci Rep. 2018 Jul 9;8:10311. doi: 10.1038/s41598-018-28628-y (PMC6037786; doi:10.1038/s41598-018-28628-y)
Supplement: Supplementary file 1 — Supplementary Information [file 41598_2018_28628_MOESM1_ESM.pdf]

Supplementary Information

MifM-instructed translation arrest involves nascent chain interactions with the exterior as well as the interior of the ribosome

Keigo Fujiwara, Koreaki Ito, and Shinobu Chiba*

Faculty of Life Sciences and Institute for Protein Dynamics, Kyoto Sangyo University, Motoyama, Kamigamo, Kita-Ku, Kyoto 603-8555, Japan

*Correspondence and requests for materials should be addressed to S.C. (schiba@cc.kyoto-su.ac.jp.)

TEL: +81-75-705-1504

Table S1. *B. subtilis* Strains

Strains	Genotype	Background	Plasmid	Ref.
SCB2639	*amyE::rbsm1-gfp-mifM35-95-lacZΩcat, rplWΩkan*			Sohmen et al., 2015
SCB2655	*amyE::rbsm1-gfp-mifM35-95-lacZΩcat, rplW(d65-69)Ωkan*			Sohmen et al., 2015
KFB203	*amyE::rbsm1-gfp-mifM35-95-lacZΩcat*	PY79	pCH1517	This study
KFB192	*amyE::rbsm1-gfp-mifM35-95(fs35-44)-lacZΩcat*	PY79	pKIG119	This study
KFB339	*amyE::rbsm1-gfp-mifM35-95(fs35-46)-lacZΩcat*	PY79	pKIG202	This study
KFB341	*amyE::rbsm1-gfp-mifM35-95(fs35-48)-lacZΩcat*	PY79	pKIG204	This study
KFB380	*amyE::rbsm1-gfp-mifM35-95(fs35-50)-lacZΩcat*	PY79	pKIG207	This study
KFB345	*amyE::rbsm1-gfp-mifM35-95(fs35-52)-lacZΩcat*	PY79	pKIG208	This study
KFB194	*amyE::rbsm1-gfp-mifM35-95(fs35-54)-lacZΩcat*	PY79	pKIG121	This study
KFB211	*amyE::rbsm1-gfp-mifM35-95(fs35-56)-lacZΩcat*	PY79	pKIG127	This study
KFB213	*amyE::rbsm1-gfp-mifM35-95(fs35-58)-lacZΩcat*	PY79	pKIG128	This study
KFB215	*amyE::rbsm1-gfp-mifM35-95(fs35-60)-lacZΩcat*	PY79	pKIG129	This study
KFB347	*amyE::rbsm1-gfp-mifM35-95(fs35-62)-lacZΩcat*	PY79	pKIG130	This study
KFB196	*amyE::rbsm1-gfp-mifM35-95(fs35-64)-lacZΩcat*	PY79	pKIG123	This study
KFB198	*amyE::rbsm1-gfp-mifM35-95(fs35-74)-lacZΩcat*	PY79	pKIG125	This study
KFB349	*amyE::rbsm1-gfp-mifM35-95(fs35-84)-lacZΩcat*	PY79	pKIG211	This study
KFB351	*amyE::rbsm1-gfp-mifM35-95(fs35-94)-lacZΩcat*	PY79	pKIG215	This study
KFB359	*amyE::rbsm1-gfp-mifM35-95(fs41-60)-lacZΩcat*	PY79	pKIG223	This study
KFB361	*amyE::rbsm1-gfp-mifM35-95(fs45-60)-lacZΩcat*	PY79	pKIG225	This study
KFB363	*amyE::rbsm1-gfp-mifM35-95(fs49-60)-lacZΩcat*	PY79	pKIG227	This study
KFB365	*amyE::rbsm1-gfp-mifM35-95(fs53-60)-lacZΩcat*	PY79	pKIG229	This study
KFB367	*amyE::rbsm1-gfp-mifM35-95(fs57-60)-lacZΩcat*	PY79	pKIG231	This study
KFB409	*amyE::rbsm1-gfp-mifM35-95(41-60GS)-lacZΩcat*	PY79	pKIG244	This study
KFB439	*amyE::rbsm1-gfp-mifM35-95(41-60 BsFtsZCTL-1)-lacZΩcat*	PY79	pKIG260	This study
KFB441	*amyE::rbsm1-gfp-mifM35-95(41-60 BsFtsZCTL-2)-lacZΩcat*	PY79	pKIG262	This study
KFB443	*amyE::rbsm1-gfp-mifM35-95(41-60 EcFtsZCTL-1)-lacZΩcat*	PY79	pKIG264	This study
KFB445	*amyE::rbsm1-gfp-mifM35-95(41-60 EcFtsZCTL-2)-lacZΩcat*	PY79	pKIG266	This study
KFB411	*amyE::rbsm1-gfp-mifM35-95(41-44GS)-lacZΩcat*	PY79	pKIG246	This study
KFB421	*amyE::rbsm1-gfp-mifM35-95(41-52GS)-lacZΩcat*	PY79	pKIG256	This study
KFB423	*amyE::rbsm1-gfp-mifM35-95(49-60GS)-lacZΩcat*	PY79	pKIG258	This study
KFB419	*amyE::rbsm1-gfp-mifM35-95(57-60GS)-lacZΩcat*	PY79	pKIG254	This study
KFB376	*amyE::rbsm1-gfp-mifM35-95-lacZΩcat, ΔrplW::kan*	KFB203	pKIG240	This study
KFB447	*amyE::rbsm1-gfp-mifM35-95-lacZΩcat, rplW(d83-94)Ωkan*	KFB203	pKIG278	This study
KFB369	*amyE::rbsm1-gfp-mifM35-95-lacZΩcat, rpmCΩkan*	KFB203	pKIG233	This study
KFB371	*amyE::rbsm1-gfp-mifM35-95-lacZΩcat, ΔrpmC::kan*	KFB203	pKIG235	This study
KFB690	*amyE::rbsm1-gfp-mifM35-95(E41A)-lacZΩcat*	PY79	pKIG529	This study
KFB691	*amyE::rbsm1-gfp-mifM35-95(Q42A)-lacZΩcat*	PY79	pKIG530	This study
KFB692	*amyE::rbsm1-gfp-mifM35-95(W43A)-lacZΩcat*	PY79	pKIG531	This study
KFB693	*amyE::rbsm1-gfp-mifM35-95(K44A)-lacZΩcat*	PY79	pKIG532	This study
KFB694	*amyE::rbsm1-gfp-mifM35-95(S45A)-lacZΩcat*	PY79	pKIG533	This study
KFB695	amyE::rbsm1-gfp-mifM35-95(K46A)-lacZΩcat	PY79	pKIG534	This study
KFB696	*amyE::rbsm1-gfp-mifM35-95(R47A)-lacZΩcat*	PY79	pKIG535	This study
KFB697	*amyE::rbsm1-gfp-mifM35-95(T48A)-lacZΩcat*	PY79	pKIG536	This study
KFB698	*amyE::rbsm1-gfp-mifM35-95(D49A)-lacZΩcat*	PY79	pKIG537	This study
KFB699	*amyE::rbsm1-gfp-mifM35-95(E50A)-lacZΩcat*	PY79	pKIG538	This study
KFB700	*amyE::rbsm1-gfp-mifM35-95(H51A)-lacZΩcat*	PY79	pKIG539	This study
KFB701	*amyE::rbsm1-gfp-mifM35-95(Q52A)-lacZΩcat*	PY79	pKIG540	This study

KFB702	*amyE::rbsm1-gfp-mifM35-95(T53A)-lacZΩcat*	PY79	pKIG541	This study
KFB703	*amyE::rbsm1-gfp-mifM35-95(V54A)-lacZΩcat*	PY79	pKIG542	This study
KFB704	*amyE::rbsm1-gfp-mifM35-95(F55A)-lacZΩcat*	PY79	pKIG543	This study
KFB705	*amyE::rbsm1-gfp-mifM35-95(H56A)-lacZΩcat*	PY79	pKIG544	This study
KFB706	*amyE::rbsm1-gfp-mifM35-95(I57A)-lacZΩcat*	PY79	pKIG545	This study
KFB707	*amyE::rbsm1-gfp-mifM35-95(N58A)-lacZΩcat*	PY79	pKIG546	This study
KFB708	*amyE::rbsm1-gfp-mifM35-95(R59A)-lacZΩcat*	PY79	pKIG547	This study
KFB709	*amyE::rbsm1-gfp-mifM35-95(T60A)-lacZΩcat*	PY79	pKIG548	This study
KFB718	*amyE::rbsm1-gfp-mifM35-95(I70A)-lacZΩcat*	PY79	pKIG567	This study
KFB770	*amyE::lacI-Pgrac-rbsm1-mifM35-95-lacZΩcat*	PY79	pKIG689	This study
KFB771	*amyE::lacI-Pgrac-rbsm1-mifM35-95(86AAAA89)-lacZΩcat*	PY79	pKIG690	This study
KFB772	*amyE::lacI-Pgrac-rbsm1-mifM41-95-lacZΩcat*	PY79	pKIG691	This study
KFB773	*amyE::lacI-Pgrac-rbsm1-mifM41-95(86AAAA89)-lacZΩcat*	PY79	pKIG692	This study
KFB768	*amyE::lacI-Pgrac-rbsm1-mifM61-95-lacZΩcat*	PY79	pKIG685	This study
KFB769	*amyE::lacI-Pgrac-rbsm1-mifM61-95(86AAAA89)-lacZΩcat*	PY79	pKIG686	This study
KFB774	*amyE::lacI-Pgrac-rbsm1-mifM66-95-lacZΩcat*	PY79	pKIG693	This study
KFB775	*amyE::lacI-Pgrac-rbsm1-mifM66-95(86AAAA89)-lacZΩcat*	PY79	pKIG694	This study
KFB776	*amyE::lacI-Pgrac-rbsm1-mifM71-95-lacZΩcat*	PY79	pKIG695	This study
KFB777	*amyE::lacI-Pgrac-rbsm1-mifM71-95(86AAAA89)-lacZΩcat*	PY79	pKIG696	This study
KFB778	*amyE::lacI-Pgrac-rbsm1-mifM76-95-lacZΩcat*	PY79	pKIG697	This study
KFB779	*amyE::lacI-Pgrac-rbsm1-mifM76-95(86AAAA89)-lacZΩcat*	PY79	pKIG698	This study
KFB794	*amyE::lacI-Pgrac-rbsm1-mifM43-95-lacZΩcat*	PY79	pKIG777	This study
KFB795	*amyE::lacI-Pgrac-rbsm1-mifM43-95(86AAAA89)-lacZΩcat*	PY79	pKIG778	This study
KFB796	*amyE::lacI-Pgrac-rbsm1-mifM45-95-lacZΩcat*	PY79	pKIG779	This study
KFB797	*amyE::lacI-Pgrac-rbsm1-mifM45-95(86AAAA89)-lacZΩcat*	PY79	pKIG780	This study
KFB798	*amyE::lacI-Pgrac-rbsm1-mifM47-95-lacZΩcat*	PY79	pKIG781	This study
KFB799	*amyE::lacI-Pgrac-rbsm1-mifM47-95(86AAAA89)-lacZΩcat*	PY79	pKIG782	This study
KFB800	*amyE::lacI-Pgrac-rbsm1-mifM49-95-lacZΩcat*	PY79	pKIG783	This study
KFB801	*amyE::lacI-Pgrac-rbsm1-mifM49-95(86AAAA89)-lacZΩcat*	PY79	pKIG784	This study

Sohmen, D. *et al*. Structure of the *Bacillus subtilis* 70S ribosome reveals the basis for species-specific stalling. *Nat. Commun.* **6**, 6941 (2015).

Table S2. Plasmids used in this study

Plasmid	Genotype	Method or ref.	Template	Primers Fw	Primers Rv
pCH746	*amyE::mifM-yidC2'-lacZΩcat*	Chiba et al., 2009			
pCH1142	*spcRΩloxP-kanR-loxP*	Kumazaki et al., 2014			
pCH1517	*amyE::rbsm1-GFP-mifM35-95-lacZΩcat*	Sohmen et al., 2015			
pCH1570	*rplWΩkanRΩrplB*	Sohmen et al., 2015			
pKIG116	*amyE::rbsm1-gfp-mifM35-95(cytosine deletion at codon 35)-lacZΩcat*	PrimeSTAR mutagenesis	pCH1517	KP1	KP2
pKIG119	*amyE::rbsm1-gfp-mifM35-95(fs35-44)-lacZΩcat*	PrimeSTAR mutagenesis	pKIG116	KP3	KP4
pKIG121	*amyE::rbsm1-gfp-mifM35-95(fs35-54)-lacZΩcat*	PrimeSTAR mutagenesis	pKIG116	KP5	KP6
pKIG123	*amyE::rbsm1-gfp-mifM35-95(fs35-64)-lacZΩcat*	PrimeSTAR mutagenesis	pKIG116	KP7	KP8
pKIG125	*amyE::rbsm1-gfp-mifM35-95(fs35-74)-lacZΩcat*	PrimeSTAR mutagenesis	pKIG116	KP9	KP10
pKIG127	*amyE::rbsm1-gfp-mifM35-95(fs35-56)-lacZΩcat*	PrimeSTAR mutagenesis	pKIG116	KP11	KP12
pKIG128	*amyE::rbsm1-gfp-mifM35-95(fs35-58)-lacZΩcat*	PrimeSTAR mutagenesis	pKIG116	KP13	KP12
pKIG129	*amyE::rbsm1-gfp-mifM35-95(fs35-60)-lacZΩcat*	PrimeSTAR mutagenesis	pKIG116	KP14	KP15
pKIG130	*amyE::rbsm1-gfp-mifM35-95(fs35-62)-lacZΩcat*	Site-directed mutagenesis	pKIG116	KP16	
pKIG202	*amyE::rbsm1-gfp-mifM35-95(fs35-46)-lacZΩcat*	Site-directed mutagenesis	pKIG116	KP17	
pKIG204	*amyE::rbsm1-gfp-mifM35-95(fs35-48)-lacZΩcat*	Site-directed mutagenesis	pKIG116	KP18	
pKIG207	*amyE::rbsm1-gfp-mifM35-95(fs35-50)-lacZΩcat*	Site-directed mutagenesis	pKIG116	KP19	
pKIG208	*amyE::rbsm1-gfp-mifM35-95(fs35-52)-lacZΩcat*	Site-directed mutagenesis	pKIG116	KP20	
pKIG211	*amyE::rbsm1-gfp-mifM35-95(fs35-84)-lacZΩcat*	Site-directed mutagenesis	pKIG116	KP21	
pKIG213	*amyE::rbsm1-gfp-mifM35-95(fs35-94, stop codon at codon 80 and 84)-lacZΩcat*	Site-directed mutagenesis	pKIG116	KP22	
pKIG215	*amyE::rbsm1-gfp-mifM35-95(fs35–94)-lacZΩcat*	Site-directed mutagenesis	pKIG213	KP23	
pKIG221	*amyE::rbsm1-gfp-mifM35-95(guanine insertion at codon 60)-lacZΩcat*	PrimeSTAR mutagenesis	pCH1517	KP14	KP15
pKIG223	*amyE::rbsm1-gfp-mifM35-95(fs41-60)-lacZΩcat*	PrimeSTAR mutagenesis	pKIG221	KP25	KP26
pKIG225	*amyE::rbsm1-gfp-mifM35-95(fs45-60)-lacZΩcat*	PrimeSTAR mutagenesis	pKIG221	KP27	KP28
pKIG227	*amyE::rbsm1-gfp-mifM35-95(fs49-60)-lacZΩcat*	PrimeSTAR mutagenesis	pKIG221	KP29	KP30
pKIG229	*amyE::rbsm1-gfp-mifM35-95(fs53-60)-lacZΩcat*	PrimeSTAR mutagenesis	pKIG221	KP31	KP32
pKIG231	*amyE::rbsm1-gfp-mifM35-95(fs57-60)-lacZΩcat*	PrimeSTAR mutagenesis	pKIG221	KP33	KP34
pKIG233	*rpmCΩkanΩrpsQ*	Gibson assembly	pCH1142	KP35	KP36
			PY79	KP37	KP38
			pCH1142	KP39	KP40
			PY79	KP41	KP42
pKIG235	*ΔrpmC::kan*	PrimeSTAR mutagenesis	pKIG233	KP43	KP44
pKIG240	*ΔrplW::kan*	PrimeSTAR mutagenesis	pCH1570	KP45	KP46
pKIG244	*amyE::rbsm1-gfp-mifM35-95(GS41-60)-lacZΩcat*	PrimeSTAR mutagenesis	pCH1517	KP47	KP48
pKIG246	*amyE::rbsm1-gfp-mifM35-95(GS41-44)-lacZΩcat*	PrimeSTAR mutagenesis	pCH1517	KP49	KP50
pKIG254	*amyE::rbsm1-gfp-mifM35-95(GS57-60)-lacZΩcat*	PrimeSTAR mutagenesis	pCH1517	KP51	KP52
pKIG256	*amyE::rbsm1-gfp-mifM35-95(GS41-52)-lacZΩcat*	PrimeSTAR mutagenesis	pCH1517	KP53	KP54
pKIG258	*amyE::rbsm1-gfp-mifM35-95(GS49-60)-lacZΩcat*	Gibson assembly	pCH1517	KP55	KP56
			pCH1517	KP57	KP58
pKIG260	*amyE::rbsm1-gfp-mifM35-95(41-60 BsFtsZCTL-1)-lacZΩcat*	PrimeSTAR mutagenesis	pCH1517	KP59	KP60
pKIG262	*amyE::rbsm1-gfp-mifM35-95(41-60 BsFtsZCTL-2)-lacZΩcat*	PrimeSTAR mutagenesis	pCH1517	KP61	KP62
pKIG264	*amyE::rbsm1-gfp-mifM35-95(41-60 EcFtsZCTL-1)-lacZΩcat*	PrimeSTAR mutagenesis	pCH1517	KP63	KP64
pKIG266	*amyE::rbsm1-gfp-mifM35-95(41-60 EcFtsZCTL-2)-lacZΩcat*	PrimeSTAR mutagenesis	pCH1517	KP65	KP66
pKIG278	*rplW(d83-94)Ωkan*	PrimeSTAR mutagenesis	pCH1570	KP67	KP68
pKIG529	*amyE::rbsm1-gfp-mifM35-95(E41A)-lacZΩcat*	Gibson assembly	pCH1517	KP69	KP56
			pCH1517	KP57	KP70
pKIG530	*amyE::rbsm1-gfp-mifM35-95(Q42A)-lacZΩcat*	Gibson assembly	pCH1517	KP71	KP56
			pCH1517	KP57	KP70
pKIG531	*amyE::rbsm1-gfp-mifM35-95(W43A)-lacZΩcat*	Gibson assembly	pCH1517	KP72	KP56
			pCH1517	KP57	KP70
pKIG532	*amyE::rbsm1-gfp-mifM35-95(K44A)-lacZΩcat*	Gibson assembly	pCH1517	KP73	KP56
			pCH1517	KP57	KP70
pKIG533	*amyE::rbsm1-gfp-mifM35-95(S45A)-lacZΩcat*	Gibson assembly	pCH1517	KP74	KP56
			pCH1517	KP57	KP75
pKIG534	*amyE::rbsm1-gfp-mifM35-95(K46A)-lacZΩcat*	Gibson assembly	pCH1517	KP76	KP56
			pCH1517	KP57	KP75
pKIG535	*amyE::rbsm1-gfp-mifM35-95(R47A)-lacZΩcat*	Gibson assembly	pCH1517	KP77	KP56
			pCH1517	KP57	KP75
pKIG536	*amyE::rbsm1-gfp-mifM35-95(T48A)-lacZΩcat*	Gibson assembly	pCH1517	KP78	KP56
			pCH1517	KP57	KP75
pKIG537	*amyE::rbsm1-gfp-mifM35-95(D49A)-lacZΩcat*	Gibson assembly	pCH1517	KP79	KP56
			pCH1517	KP57	KP80
pKIG538	*amyE::rbsm1-gfp-mifM35-95(E50A)-lacZΩcat*	Gibson assembly	pCH1517	KP81	KP56
			pCH1517	KP57	KP80
pKIG539	*amyE::rbsm1-gfp-mifM35-95(H51A)-lacZΩcat*	Gibson assembly	pCH1517	KP82	KP56
			pCH1517	KP57	KP80
pKIG540	*amyE::rbsm1-gfp-mifM35-95(Q52A)-lacZΩcat*	Gibson assembly	pCH1517	KP83	KP56
			pCH1517	KP57	KP80

pKIG541	*amyE::rbsm1-gfp-mifM35-95(T53A)-lacZΩcat*	Gibson assembly	pCH1517	KP84	KP56
			pCH1517	KP57	KP85
pKIG542	*amyE::rbsm1-gfp-mifM35-95(V54A)-lacZΩcat*	Gibson assembly	pCH1517	KP86	KP56
			pCH1517	KP57	KP85
pKIG543	*amyE::rbsm1-gfp-mifM35-95(F55A)-lacZΩcat*	Gibson assembly	pCH1517	KP87	KP56
			pCH1517	KP57	KP85
pKIG544	*amyE::rbsm1-gfp-mifM35-95(H56A)-lacZΩcat*	Gibson assembly	pCH1517	KP88	KP56
			pCH1517	KP57	KP85
pKIG545	*amyE::rbsm1-gfp-mifM35-95(I57A)-lacZΩcat*	Gibson assembly	pCH1517	KP89	KP56
			pCH1517	KP57	KP90
pKIG546	*amyE::rbsm1-gfp-mifM35-95(N58A)-lacZΩcat*	Gibson assembly	pCH1517	KP91	KP56
			pCH1517	KP57	KP90
pKIG547	*amyE::rbsm1-gfp-mifM35-95(R59A)-lacZΩcat*	Gibson assembly	pCH1517	KP92	KP56
			pCH1517	KP57	KP90
pKIG548	*amyE::rbsm1-gfp-mifM35-95(T60A)-lacZΩcat*	Gibson assembly	pCH1517	KP93	KP56
			pCH1517	KP57	KP90
pKIG567	*amyE::rbsm1-gfp-mifM35-95(I70A)-lacZΩcat*	Site-directed mutagenesis	pCH1517	KP94	
pKIG367	*amyE:: lacI-PgracΩcat*	Gibson assembly	pDG1662	KP95	KP96
			pHT01	KP97	KP98
pKIG406	*amyE::lacI–Pgrac–rbsm1–gfp–mifM–lacZΩcat*	Gibson assembly	pKIG367	KP99	KP100
			pKIG40	KP101	KP102
pKIG681	*amyE::lacI-Pgrac-rbsm1-gfp-mifM(86AAAA89)-lacZΩcat*	Gibson assembly	pKIG406	KP103	KP104
			pKIG406	KP105	KP106
pKIG685	*amyE::lacI-Pgrac-rbsm1-mifM61-95-lacZΩcat*	Gibson assembly	pKIG406	KP107	KP104
			pKIG406	KP105	KP108
pKIG686	*amyE::lacI-Pgrac-rbsm1-mifM61-95(86AAAA89)-lacZΩcat*	Gibson assembly	pKIG681	KP107	KP104
			pKIG681	KP105	KP108
pKIG689	*amyE::lacI-Pgrac-rbsm1-mifM35-95-lacZΩcat*	Gibson assembly	pKIG406	KP105	KP109
			pKIG406	KP110	KP104
pKIG690	*amyE::lacI-Pgrac-rbsm1-mifM35-95(86AAAA89)-lacZΩcat*	Gibson assembly	pKIG406	KP105	KP109
			pKIG681	KP110	KP104
pKIG691	*amyE::lacI-Pgrac-rbsm1-mifM41-95-lacZΩcat*	Gibson assembly	pKIG406	KP105	KP109
			pKIG406	KP111	KP104
pKIG692	*amyE::lacI-Pgrac-rbsm1-mifM41-95(86AAAA89)-lacZΩcat*	Gibson assembly	pKIG406	KP105	KP109
			pKIG681	KP111	KP104
pKIG693	*amyE::lacI-Pgrac-rbsm1-mifM66-95-lacZΩcat*	Gibson assembly	pKIG406	KP105	KP109
			pKIG406	KP112	KP104
pKIG694	*amyE::lacI-Pgrac-rbsm1-mifM66-95(86AAAA89)-lacZΩcat*	Gibson assembly	pKIG406	KP105	KP109
			pKIG681	KP112	KP104
pKIG695	*amyE::lacI-Pgrac-rbsm1-mifM71-95-lacZΩcat*	Gibson assembly	pKIG406	KP105	KP109
			pKIG406	KP113	KP104
pKIG696	*amyE::lacI-Pgrac-rbsm1-mifM71-95(86AAAA89)-lacZΩcat*	Gibson assembly	pKIG406	KP105	KP109
			pKIG681	KP113	KP104
pKIG697	*amyE::lacI-Pgrac-rbsm1-mifM76-95-lacZΩcat*	Gibson assembly	pKIG406	KP105	KP109
			pKIG406	KP114	KP104
pKIG698	*amyE::lacI-Pgrac-rbsm1-mifM76-95(86AAAA89)-lacZΩcat*	Gibson assembly	pKIG406	KP105	KP109
			pKIG681	KP114	KP104
pKIG777	*amyE::lacI-Pgrac-rbsm1-mifM43-95-lacZΩcat*	Gibson assembly	pKIG406	KP105	KP109
			pKIG681	KP122	KP104
pKIG778	*amyE::lacI-Pgrac-rbsm1-mifM43-95(86AAAA89)-lacZΩcat*	Gibson assembly	pKIG406	KP105	KP109
			pKIG681	KP122	KP104
pKIG779	*amyE::lacI-Pgrac-rbsm1-mifM45-95-lacZΩcat*	Gibson assembly	pKIG406	KP105	KP109
			pKIG681	KP123	KP104
pKIG780	*amyE::lacI-Pgrac-rbsm1-mifM45-95(86AAAA89)-lacZΩcat*	Gibson assembly	pKIG406	KP105	KP109
			pKIG681	KP123	KP104
pKIG781	*amyE::lacI-Pgrac-rbsm1-mifM47-95-lacZΩcat*	Gibson assembly	pKIG406	KP105	KP109
			pKIG681	KP124	KP104
pKIG782	*amyE::lacI-Pgrac-rbsm1-mifM47-95(86AAAA89)-lacZΩcat*	Gibson assembly	pKIG406	KP105	KP109
			pKIG681	KP124	KP104
pKIG783	*amyE::lacI-Pgrac-rbsm1-mifM49-95-lacZΩcat*	Gibson assembly	pKIG406	KP105	KP109
			pKIG681	KP125	KP104
pKIG784	*amyE::lacI-Pgrac-rbsm1-mifM49-95(86AAAA89)-lacZΩcat*	Gibson assembly	pKIG406	KP105	KP109
			pKIG681	KP125	KP104

Chiba, S., Lamsa, A., & Pogliano, K. A ribosome-nascent chain sensor of membrane protein biogenesis in *Bacillus subtilis*. *The EMBO J*. **28**, 3461-75 (2009).

Kumazaki, K. *et al*. Structural basis of Sec-independent membrane protein insertion by YidC. *Nature* **509**, 516-20 (2014).

Sohmen, D. *et al*. Structure of the *Bacillus subtilis* 70S ribosome reveals the basis for species-specific stalling. *Nat. Commun*. **6**, 6941 (2015).

Table S3. Oligonucleotides used in this study

#	DNA sequence
KP1	5'-AGGATCCGGGAGTGCCGTGCGGGCG-3'
KP2	5'-GCACTCCCGGATCCTGTTGCACCCCG-3'
KP3	5'-ATGGAAATTCAAAACGAACAGATGAA-3'
KP4	5'-GTTTTGAATTTCCATTGCTCGCCCGC-3'
KP5	5'-GACGGTCGTTTCACATTAACCGAACA-3'
KP6	5'-TGTGAAACGACCGTCTGATGTTCATC-3'
KP7	5'-TCTTATTTATTATATATCATCGCATT-3'
KP8	5'-ATATAATAAATAAGAAAGTCTGTTCG-3'
KP9	5'-TTGGATATCGTAAAGTCTTCCGCATG-3'
KP10	5'-CTTTACGATATCCAAGTTGTAATGCG-3'
KP11	5'-CAGACGGTCTTTCACCATTAACCGAACAGACTTTCTTATT-3'
KP12	5'-GTGAAAGACCGTCTGATGTTCATCTGTTCGTTTTG-3'
KP13	5'-CAGACGGTCTTTCACATTAACCCGAACAGACTTTCTTATT-3'
KP14	5'-CACATTAACCGAACAGGACTTTCTTATTATTATAT-3'
KP15	5'-TGTTCGGTTAATGTGAAAGACCGTCTGATGTTCAT-3'
KP16	5'-CACATTAACCGAACAGACTTTCCTTATTATTATATATCAT-3'
KP17	5'-GCGAGCAATGGAAATCAAAACCGAACAGATGAACATCAGACG-3'
KP18	5'-AATGGAAATCAAAACGAACAGGATGAACATCAGACGGTCTTT-3'
KP19	5'-AATCAAAACGAACAGATGAACCATCAGACGGTCTTTCACATT-3'
KP20	5'-AACGAACAGATGAACATCAGAACGGTCTTTCACATTAACCGA-3'
KP21	5'-CGTAAAGTCTTCCGCATGGATTCGCCTGTGGAACGATGAGGAAGACGCC-3'
KP22	5'-TCTTCTTTTTAAGATCCAAGCTTACTAGTACCA-3'
KP23	5'-CGTAAAGTCTTCCGCATGGATTCGCCTGTGGACGATGAGGAAGACGCCG-3'
KP24	5'-CACATTAACCGAACAGGACTTTCTTATTATTATAT-3'
KP25	5'-TGCGGGCAGCAATGGAAATCAAAACGAACAGAT-3'
KP26	5'-TCCATTGCTGCCCGCACGGCACTCCCGGGATCC-3'
KP27	5'-ATGGAAACAAAACGAACAGATGAACATCAGACG-3'
KP28	5'-TCGTTTTGTTTCCATTGCTCGCCCGCACGGCAC-3'
KP29	5'-ACGAACAATGAACATCAGACGGTCTTTCACATT-3'
KP30	5'-ATGTTCATTGTTCGTTTTGATTTCCATTGCTCG-3'
KP31	5'-ACATCAGCGGTCTTTCACATTAACCGAACAGGA-3'
KP32	5'-AAAGACCGCTGATGTTCATCTGTTCGTTTTGAT-3'
KP33	5'-CTTTCACTTAACCGAACAGGACTTTCTTATTAT-3'
KP34	5'-TCGGTTAAGTGAAAGACCGTCTGATGTTCATCT-3'
KP35	5'-TAATCAAGAGGCGCCAATTCCATGCAAGCTTGGCGTAATC-3'
KP36	5'-CTTAGCTGTGTGGATCGTAAGAGCTCGAATTCCTGCAGCT-3'
KP37	5'-TTACGATCCACACAGCTAAGC-3'
KP38	5'-TTATTTATTAGCAGCAATTTCTCTTTCACG-3'
KP39	5'-AAATTGCTGCTAATAAATAATAAATACTGTAGAAAAGAGG-3'
KP40	5'-CCTCAGTGATTATTTATTAGCTAAAACAATTCATCCAGTA-3'
KP41	5'-CTAATAAATAATCACTGAGGGGAGGT-3'
KP42	5'-GAATTGGCGCCTCTTGATTAGA-3'
KP43	5'-AAGCTAATAAATACTGTAGAAAAGAGGAAGGAA-3'
KP44	5'-AGTATTTATTAGCTTTCATTTGATTCACCACCA-3'
KP45	5'-TGCATAAATACTGTAGAAAAGAGGAAGGAAATA-3'
KP46	5'-TACAGTATTTATGCAAGCACCTCCTCTACTTTT-3'

KP47 5'-TGGTGGAGGATCCGGAGGTGGTTCAGGTGGCGGCTCTGGCGACTTTCTTATTATTATATATCATC-3'

KP48 5'-CCACCTCCGGATCCTCCACCAGAACCACCGCCTGAGCCGCCGCCCGCACGGCACTCCCGGG-3'

KP49 5'-GCGGTGGTTCTGGTTCAAAACGAACAGATGAACATC-3'

KP50 5'-GAACCAGAACCACCGCCCGCACGGCACTCCCGGGAT-3'

KP51 5'-ACGGTGGTTCTGGTGACTTTCTTATTATTATATATC-3'

KP52 5'-TCACCAGAACCACCGTGAAAGACCGTCTGATGTTCA-3'

KP53 5'-AGGCGGTGGTTCTGGTGGAGGATCCGGAACGGTCTTTCACATTAACCGA-3'

KP54 5'-CTCCACCAGAACCACCGCCTGAGCCGCCGCCCGCACGGCACTCCCGGGA-3'

KP55 5'-CGGAGGTGGTTCAGGTGGCGGCTCTGGCGACTTTCTTATTATTATATATCATC-3'

KP56 5'-ACGCGGTCATCAATCATACC-3'

KP57 5'-GGTATGATTGATGACCGCGT-3'

KP58 5'-CGCCACCTGAACCACCTCCGGATCCTCCTGTTCGTTTTGATTTCCATTGCTCG-3'

KP59 5'-GAAGCCTCAGCGTCCAAGCTTAAATCAAAGCATCAAAACAGACTTTCTTATTATTATATATCATC-3'

KP60 5'-AAGCTTGGACGCTGAGGCTTCGTCACGTCCTTCTCTTGTTCGCCCGCACGGCACTCCCGGG-3'

KP61 5'-ACCTCAGCAGCAGAACACAGTAAGCCGTCATACTTCACAGGACTTTCTTATTATTATATATCATC-3'

KP62 5'-ACTGTGTTCTGCTGCTGAGGTTCCTCACGTTTTGGCTCACGGCCCGCACGGCACTCCCGGG-3'

KP63 5'-TCTGGTGACCAATAAGCAGGTTCAGCAGCCAGTGATGGATGACTTTCTTATTATTATATATCATC-3'

KP64 5'-ACCTGCTTATTGGTCACCAGAGTGATTTCAGGACGTTTGTCGCCCGCACGGCACTCCCGGG-3'

KP65 5'-GGTTGCTAAAGTCGTGAATGACAATGCGCCGCAAACTGCGGACTTTCTTATTATTATATATCATC-3'

KP66 5'-TCATTCACGACTTTAGCAACCGGCTTCTGCTCCTGGGTCAGGCCCGCACGGCACTCCCGGG-3'

KP67 5'-CGTAAAATAAATACTGTAGAAAAGAGGAAGGAA-3'

KP68 5'-AGTATTTATTTTACGATCGCTTTTCTGCGACGG-3'

KP69 5'-CCCGGGAGTGCCGTGCGGGCGCACAATGGAAATCAAAACGAAC-3'

KP70 5'-GCCCGCACGGCACTCCCGGGATCCTGTTGC-3'

KP71 5'-CCCGGGAGTGCCGTGCGGGCGAGGCATGGAAATCAAAACGAACAGA-3'

KP72 5'-CCCGGGAGTGCCGTGCGGGCGAGCAAGCAAAATCAAAACGAACAGATGA-3'

KP73 5'-CCCGGGAGTGCCGTGCGGGCGAGCAATGGGCATCAAAACGAACAGATGAACA-3'

KP74 5'-GTGCGGGCGAGCAATGGAAAGCAAAACGAACAGATGAACATCA-3'

KP75 5'-TTTCCATTGCTCGCCCGCACGGCA-3'

KP76 5'-GTGCGGGCGAGCAATGGAAATCAGCACGAACAGATGAACATCAGAC-3'

KP77 5'-GTGCGGGCGAGCAATGGAAATCAAAAGCAACAGATGAACATCAGACGGT-3'

KP78 5'-GTGCGGGCGAGCAATGGAAATCAAAACGAGCAGATGAACATCAGACGGTCTT-3'

KP79 5'-AATGGAAATCAAAACGAACAGCAGAACATCAGACGGTCTTTCA-3'

KP80 5'-TGTTCGTTTTGATTTCCATTGCTCGCCCGC-3'

KP81 5'-AATGGAAATCAAAACGAACAGATGCACATCAGACGGTCTTTCACAT-3'

KP82 5'-AATGGAAATCAAAACGAACAGATGAAGCACAGACGGTCTTTCACATTAA-3'

KP83 5'-AATGGAAATCAAAACGAACAGATGAACATGCAACGGTCTTTCACATTAACCG-3'

KP84 5'-AACGAACAGATGAACATCAGGCAGTCTTTCACATTAACCGAAC-3'

KP85 5'-CTGATGTTCATCTGTTCGTTTTGA-3'

KP86 5'-AACGAACAGATGAACATCAGACGGCATTTCACATTAACCGAACAGA-3'

KP87 5'-AACGAACAGATGAACATCAGACGGTCGCACACATTAACCGAACAGACTT-3'

KP88 5'-AACGAACAGATGAACATCAGACGGTCTTTGCAATTAACCGAACAGACTTTCT-3'

KP89 5'-AACATCAGACGGTCTTTCACGCAAACCGAACAGACTTTCTTAT-3'

KP90 5'-GTGAAAGACCGTCTGATGTTCATCTGTTCG-3'

KP91 5'-AACATCAGACGGTCTTTCACATTGCACGAACAGACTTTCTTATTAT-3'

KP92 5'-AACATCAGACGGTCTTTCACATTAACGCAACAGACTTTCTTATTATTAT-3'

KP93 5'-AACATCAGACGGTCTTTCACATTAACCGAGCAGACTTTCTTATTATTATATA-3'

KP94 5'-CTTATTATTATATATCATCGCGCGACAACTTGGATACGTAA-3'

KP95	5'-GCGTCCATGGAGATCCGATCAGACCAGTTTTTAAT-3'
KP96	5'-TGTGAGTTAAGGCCTGATCCTAGAAGCTTATCGAA-3'
KP97	5'-AGGCCTTAACTCACATTAAT-3'
KP98	5'-GATCTCCATGGACGCGTGAC-3'
KP99	5'-CGATCAGACCAGTTTTTAATTTG-3'
KP100	5'-TTAATTGGGAATTGTTATCC-3'
KP101	5'-GGATAACAATTCCCAATTAAGGAGGAGGATGTGATGACAA-3'
KP102	5'-CAAATTAAAAACTGGTCTGATCG-3'
KP103	5'-AATTCGCCTGTGAACGCAGCAGCAGCAGCCGGTTCTCTTCTTTTAGGATC-3'
KP104	5'-GATTATCAAAAAGGATCTTCACC-3'
KP105	5'-GGTGAAGATCCTTTTTGATAATC-3'
KP106	5'-GTTCACAGGCGAATTCATGCGGAAGACTTTACG-3'
KP107	5'-TGTGATGACAGACTTTCTTATTATTATATATCATCGCATT-3'
KP108	5'-TAAGAAAGTCTGTCATCACATCCTCCTCCT-3'
KP109	5'-CATCACATCCTCCTCCTTAATTGGG-3'
KP110	5'-CCCAATTAAGGAGGAGGATGTGATGACACGGGAGTGCCGTGCGGGCGAG-3'
KP111	5'-CCCAATTAAGGAGGAGGATGTGATGACAGAGCAATGGAAATCAAAACGA-3'
KP112	5'-CCCAATTAAGGAGGAGGATGTGATGACAATATATCATCGCATTACAACT-3'
KP113	5'-CCCAATTAAGGAGGAGGATGTGATGACAACAACTTGGATACGTAAAGTC-3'
KP114	5'-CCCAATTAAGGAGGAGGATGTGATGACAAAAGTCTTCCGCATGAATTCG-3'
KP115	5'-TAACTTTAAGAAGGAGGGAGATATACCAATGACAATGTTTGTGGGATC-3'
KP116	5'-TAACTTTAAGAAGGAGGGAGATATACCAATGATGATGCGGGAGTGCCGTGCGGGCGAG-3'
KP117	5'-TAACTTTAAGAAGGAGGGAGATATACCAATGATGATGGAGCAATGGAAATCAAAACGA-3'
KP118	5'-TAACTTTAAGAAGGAGGGAGATATACCAATGATGATGGACTTTCTTATTATTATATATCATCGC-3'
KP119	5'-CAGGTCCTCTTCGGAGATGAGTTTCTGTTCTAAAAGAAGAGAACCGGC-3'
KP120	5'-GAAATTAATACGACTCACTATAGGGAGACCACAACGGTTTCCCTCTAGAAATAATTTTGTTTAACTTTAAGAAGGAG-3'
KP121	5'-ATATGGATCCTTATTACAGGTCCTCTTCGGAGATGAGTTTCTGTTC-3'
KP122	5'-CCCAATTAAGGAGGAGGATGTGATGACATGGAAATCAAAACGAACAGAT-3'
KP123	5'-CCCAATTAAGGAGGAGGATGTGATGACATCAAAACGAACAGATGAACAT-3'
KP124	5'-CCCAATTAAGGAGGAGGATGTGATGACACGAACAGATGAACATCAGACG-3'
KP125	5'-CCCAATTAAGGAGGAGGATGTGATGACAGATGAACATCAGACGGTCTTT-3'

Table S4. Templates used in vitro translation

Gene name	1st PCR template	1st fw primer	1st rv primer	2nd fw primer	2nd rv primer
gfp-mifM35-95-myc	pCH1517	KP115	KP119	KP120	KP121
gfp-mifM35-95(fs41-60)-myc	pKIG223	KP115	KP119	KP120	KP121
gfp-mifM35-95(GS41-60)-myc	pKIG244	KP115	KP119	KP120	KP121
mifM35-95-myc	pCH746	KP116	KP119	KP120	KP121
mifM35-95(I70A)-myc	pKIG567	KP116	KP119	KP120	KP121
mifM41-95-myc	pCH746	KP117	KP119	KP120	KP121
mifM41-95(I70A)-myc	pKIG567	KP117	KP119	KP120	KP121
mifM61-95-myc	pCH746	KP118	KP119	KP120	KP121
mifM61-95(I70A)-myc	pKIG567	KP118	KP119	KP120	KP121

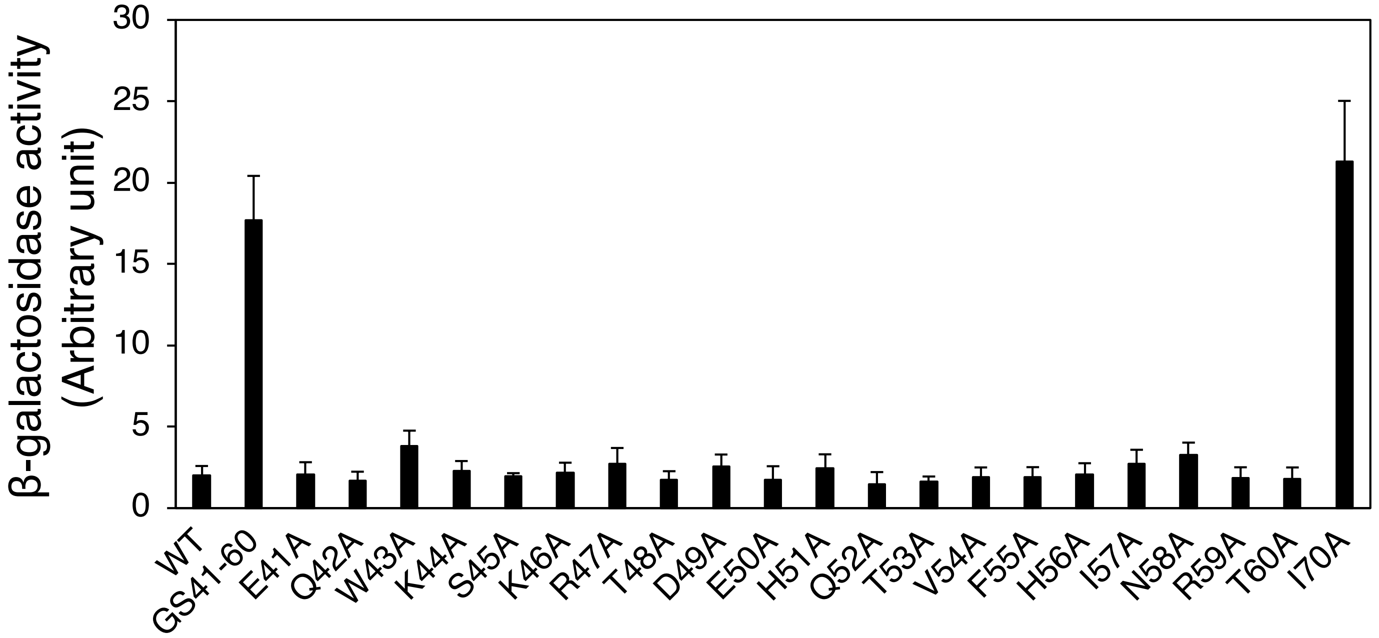

Figure S1. Alanine scanning mutagenesis of the MifM segment 41-60 under the context of the gfp-*mifM-lacZ* gene fusion. β-galactosidase activity (mean±s.d., n=3) of cells expressing the wild-type and mutant derivatives of the fusion gene. The mutations introduced are indicated below the graph. The results with the GS41-60 and the I70A derivatives are also presented as controls showing β-galactosidase activities obtained for the partially arrest-defective fusion proteins.

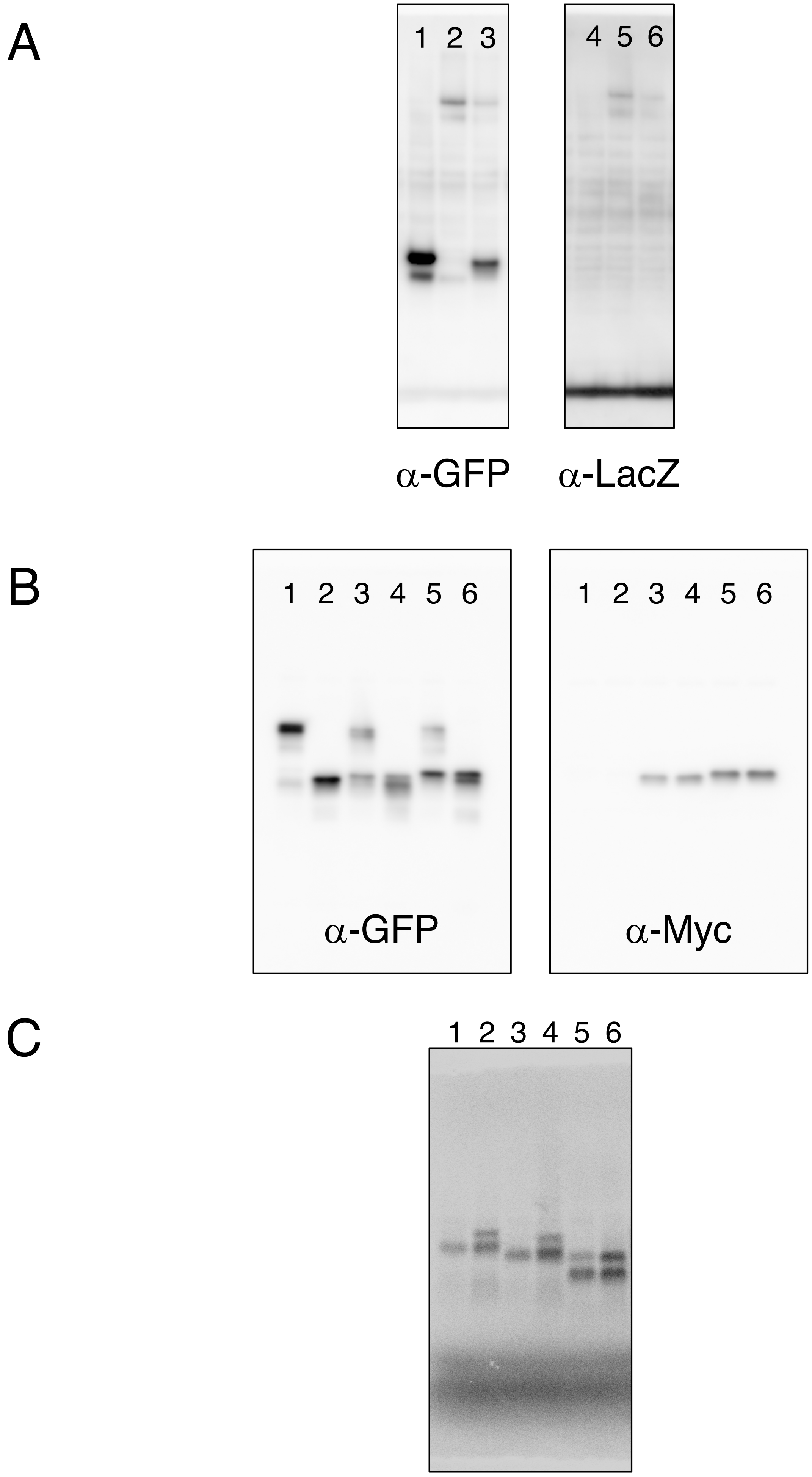

Figure S2. Uncropped images of the gel and membranes displayed in the Fig. 2 (A), Fig. 4 (B), and Fig. 5B (C). Lane numbers described in each figure legends are included in each image.
